# Supplementary figures and images for: High-level visual prediction errors in early visual cortex
Source: PLoS Biol. 2024 Nov 11;22(11):e3002829. doi: 10.1371/journal.pbio.3002829 (PMC11554119; doi:10.1371/journal.pbio.3002829)

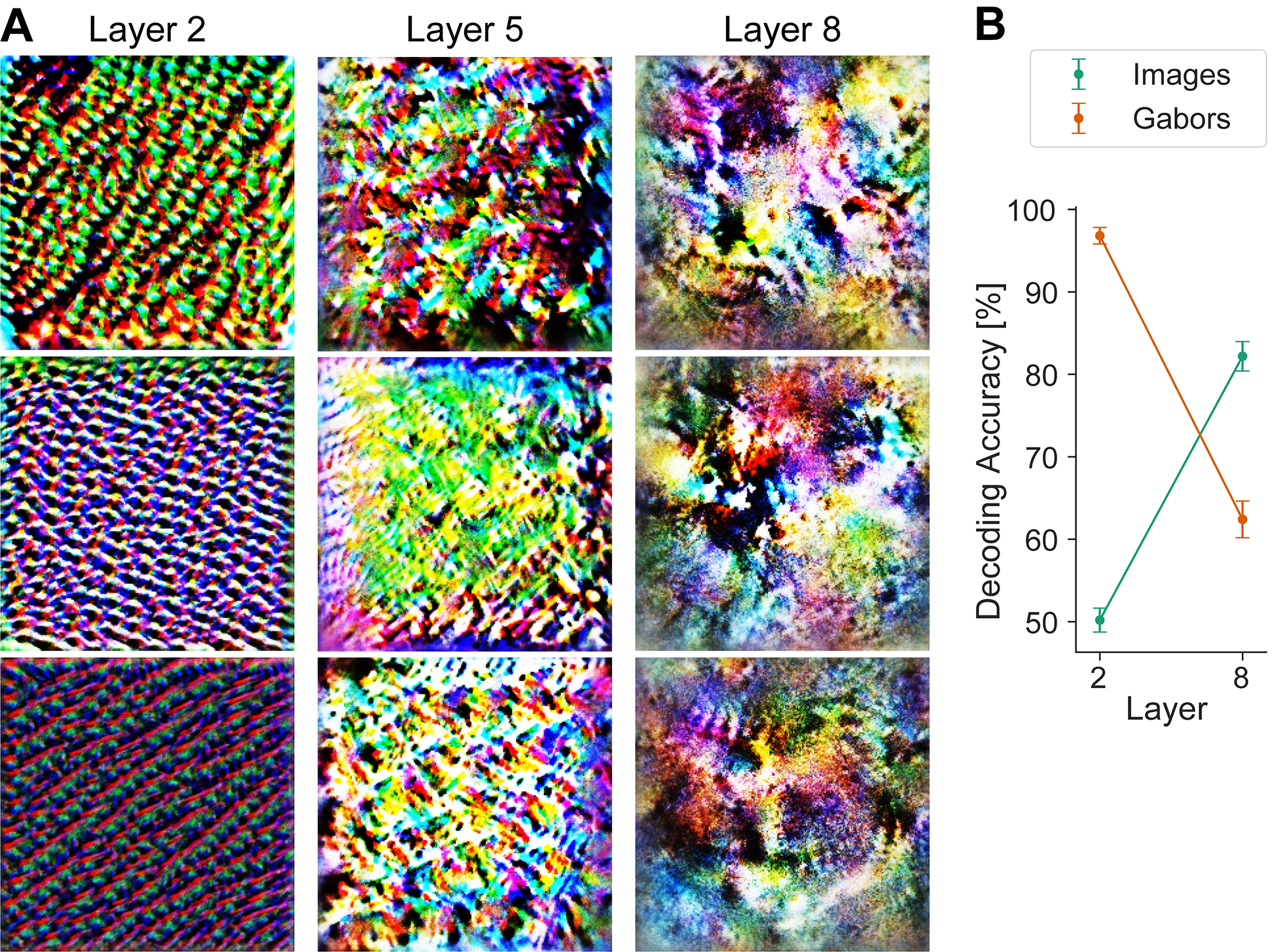

Supplement: S1 Fig — (A) Feature visualization using maximal activation. In brief, starting from random noise, an image was optimized to maximally activate a particular channel of a layer. The optimization function was the negative of the spatial activation of that layer and channel (for the code and more details, see: https://github.com/KietzmannLab/lucid-kietzmannlab). From the resulting images, maximally driving the channel responses of the layer of interest, we can thus qualitatively interpret the kinds of features represented in that layer. Depicted are example images maximally driving activation of 3 channels (y axis) of layers 2, 5, and 8 (x axis). Images on the left are characterized by repeating clear orientations, thus suggesting that layer 2 represents low-level, Gabor-like features. Layer 5, in the center column, contains larger scale orientation features, but also textures. On the right side, image exemplars maximally driving layer 8, reflect more high-level visual features, such as abstract, irregular patterns and textures. Thus, the feature visualization results suggest 2 primary conclusions. One, layer 2 does reflect typical low-level visual features, such as orientation. Second, layer 8 reflects higher level visual features compared to previous layers, most notably complex patterns and irregular textures, with little low-level visual feature representation (e.g., no evidence of orientation tuning). Therefore, the feature visualization results support our assumption that layer 2 largely reflects low-level and layer 8 high-level visual features. (B) Decoding of object image category and Gabor orientation from DNN layer activations. If the qualitative interpretation above is correct, we would expect that layer 2 contains significantly more low-level visual feature information compared to layer 8. In contrast, we would expect that more category information is available in layer 8 representations. We tested these hypotheses by performing 2 decoding analyses. First, we extrac [file pbio.3002829.s001.tif]

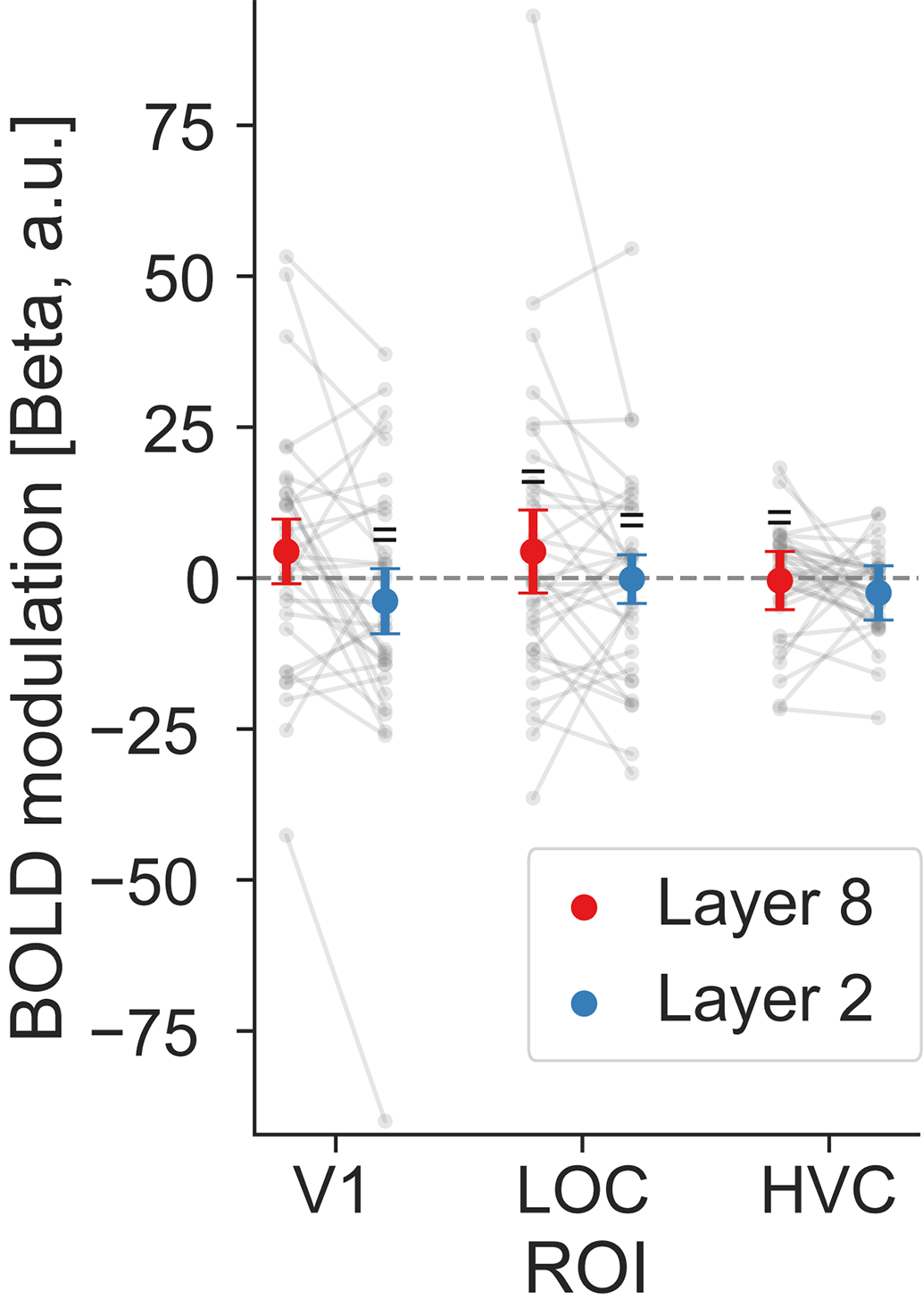

Supplement: S2 Fig — Control ROI analysis using stimulus uninformative voxels in early visual (V1), intermediate (LOC), and higher visual cortex (HVC; encompassing occipito-temporal sulcus and fusiform cortex). ROI masks were defined using the same procedure as outlined in the region of interest (ROI) analysis paragraph of the Materials and methods sections, except for that voxel selection was performed by choosing the least informative (i.e., lowest decoding performance) voxels for object identity decoding during the localizer runs. No modulation of neural responses by high-level (layer 8) or low-level (layer 2) surprise was observed in any ROI containing voxels uninformative about the stimuli. These results further suggest that the modulation of visual responses by high-level surprise (Fig 5) is specific to stimulus-selective voxels and not an unspecific global surprise signal. Error bars indicate the 95% within-subject confidence intervals. Gray dots denote individual subjects. = BF10 < 1/3. Data and code that support these findings are available at: https://doi.org/10.34973/8e49-2012. (TIF) [file pbio.3002829.s002.tif]

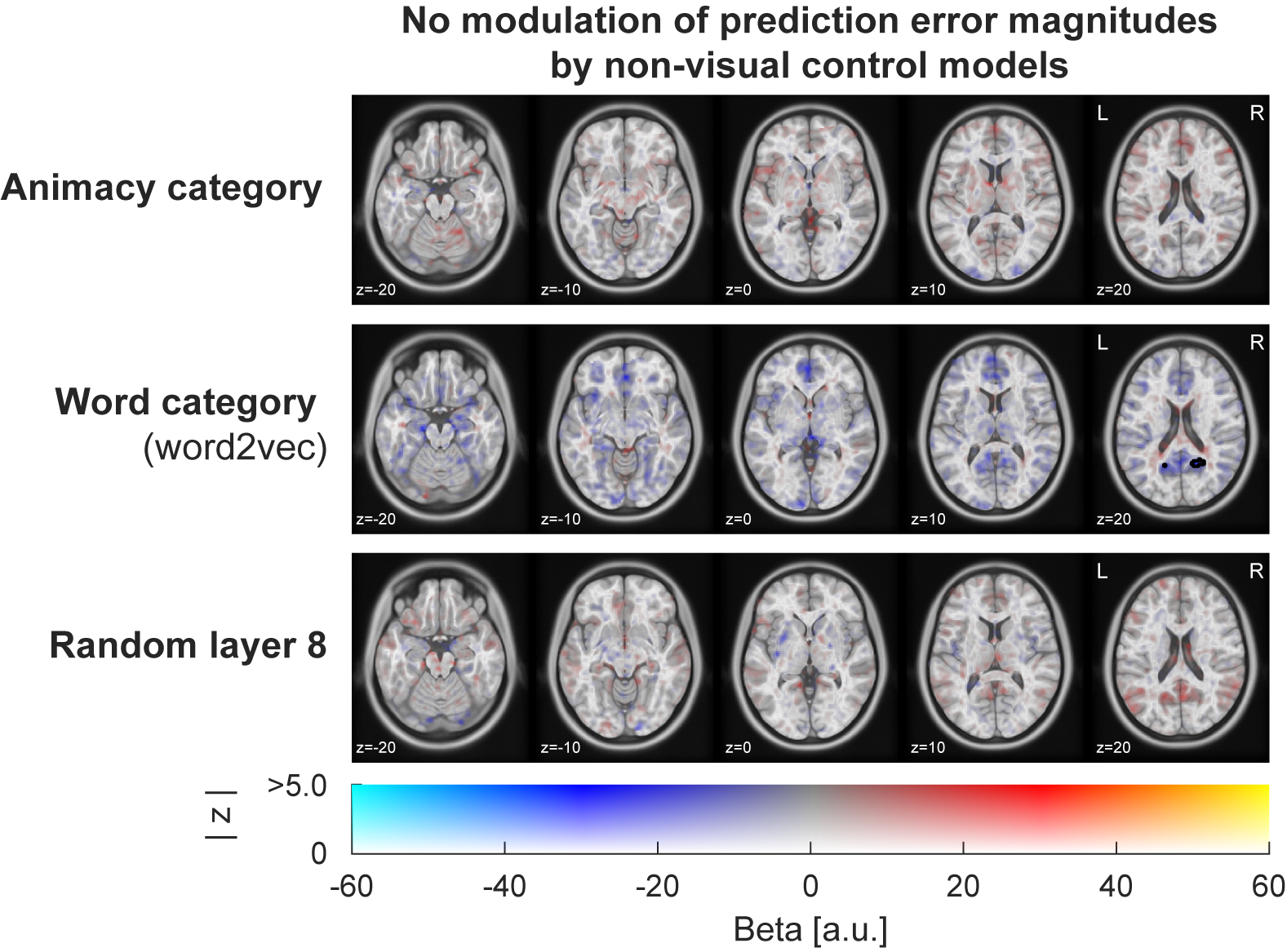

Supplement: S3 Fig — Whole-brain results assessing the modulation of surprise responses as a function of feature dissimilarity indexed by animacy category (top row), word-level semantic surprise (middle row), and a random (i.e., untrained) but otherwise identical visual DNN instance (bottom row). Results show no reliable modulation by any of these control models anywhere in cortex, except for a small negative modulation by word category surprise (word2vec) in precuneus cortex, outside of stimulus-driven voxels. Thus, unexpected stimuli of a similar semantic category as the expected stimulus may elicit larger BOLD responses. This modulation could reflect an increased requirement for processing resources to distinguish different exemplars of the same category, albeit its small size and localization to voxels in superior parts of precuneus cortex, that were not stimulus driven during the localizer run, makes an interpretation challenging. Color indicates the beta parameter estimate of the parametric modulation, with red and yellow representing increased responses. Black outlines denote statistically significant clusters (GRF cluster corrected). Data and code that support these findings are available at: https://doi.org/10.34973/8e49-2012. (TIF) [file pbio.3002829.s003.tif]

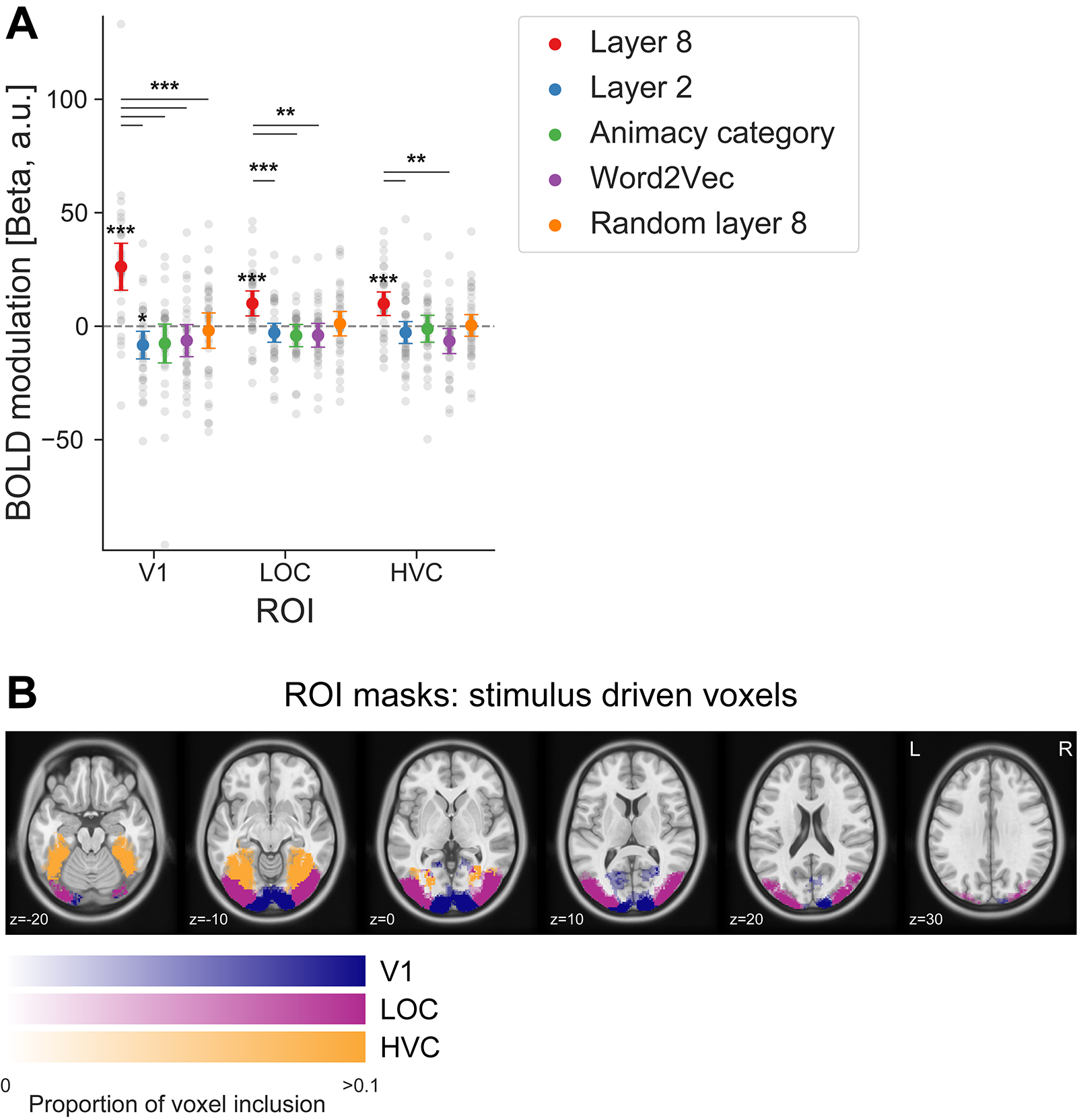

Supplement: S4 Fig — (A) Control ROI analysis using all stimulus driven within the anatomically defined V1, LOC, and HVC masks. For ROI mask creation, stimulus driven was defined as a significant activation (z > 3.1) of voxels by the presentation of the object images during the independent localizer run. Thus, compared to the ROI masks used in the main analyses, these ROI masks are larger, encompassing all visually driven voxels within the ROIs. Average mask size in voxels: V1 = 1,254, LOC = 2,443, HVC = 1,383. Results show that prediction error magnitudes are best explained by high-level visual feature dissimilarity across all 3 ROIs. Error bars indicate the 95% within-subject confidence intervals. Gray dots denote individual subjects. P values are FDR corrected. *** p < 0.001, ** p < 0.01. (B) Voxels included in the ROI masks using all stimulus-driven voxels within the anatomically defined masks. Color indicates the ROI: Blue = V1, Purple = LOC, Orange = HVC. Notably, the resulting masks are significantly larger and extend further anterior in HVC compared to the original decoding-based masks. Opacity indicates the proportion of participants whose individual masks included the voxel. For visualization, full opacity corresponds to a proportion of 0.1, with voxel inclusion proportions reaching up to 1. Data and code that support these findings are available at: https://doi.org/10.34973/8e49-2012. (TIF) [file pbio.3002829.s004.tif]

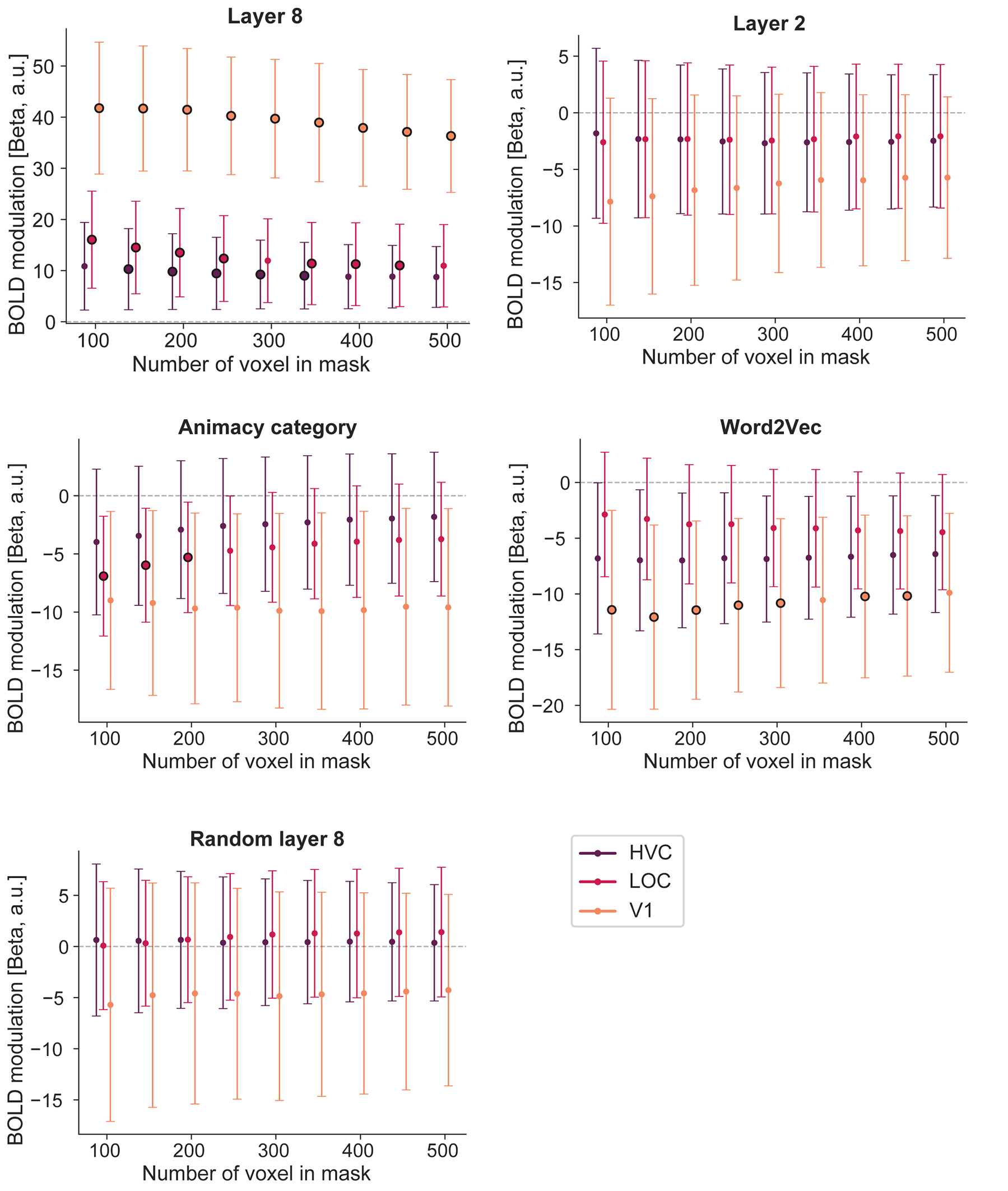

Supplement: S5 Fig — Modulation of surprise as a function of high-level (layer 8; first panel), low-level (layer 2; second panel), animacy category (third panel), word level (word2vec; fourth panel), and random model (last panel) surprise. Reliable modulations of prediction error magnitudes were found for high-level visual surprise across all tested ROI sizes (100–500 voxel) in V1, as well as most mask sizes in LOC (100–250 and 350–450 voxel) and TOFC (150–350 voxel). No statistically significant modulation was found for low-level visual surprise or the random layer 8 model for any ROI or mask size. A negative modulation of BOLD responses was found for word level (word2vec) surprise in V1 for several ROI masks (150–300 and 400–450 voxel) and 3 small mask sizes in LOC (100–200 voxel) for animacy category. Thus, overall results closely match the results reported in Fig 6 for most mask sizes, confirming that the observed results are largely robust to variations in ROI size. Error bars depict 95% confidence intervals. Data points with black outline indicate statistical significance at p < 0.05 (FDR corrected for the number of ROIs and models) compared to zero (i.e., no modulation). Data and code that support these findings are available at: https://doi.org/10.34973/8e49-2012. (TIF) [file pbio.3002829.s005.tif]

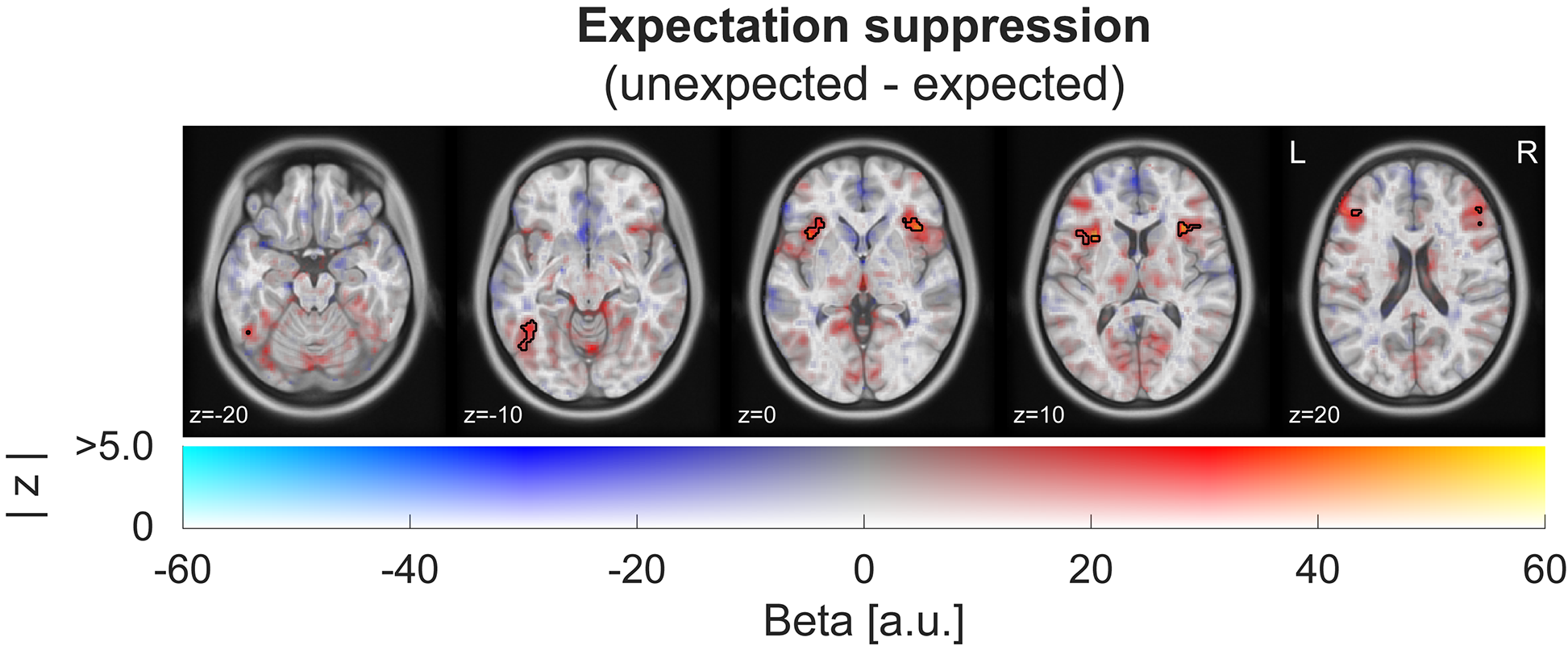

Supplement: S6 Fig — Generic prediction errors (unexpected–expected) were calculated from the voxel-wise GLM including regressors for expected and unexpected appearances of the image stimuli, as well as the parametric modulators. Here, we depict the contrast unexpected–expected, thus indexing differences in neural responses contingent on whether the stimulus was expected or unexpected. Color indicates the beta parameter estimate, with red and yellow representing increased responses to unexpected stimuli. Black outlines denote statistically significant clusters (GRF cluster corrected). Significant clusters can be seen in visual cortex, particularly in temporal occipital fusiform cortex, anterior insula, and inferior frontal gyrus. Additional significant cluster, not visible here, were found in superior parietal lobule, paracingulate gyrus, and supplementary motor cortex; see S1 Table for details. These areas closely match previous reports of prediction error responses [10,42]. The data that support these findings are available at: https://doi.org/10.34973/8e49-2012. (TIF) [file pbio.3002829.s006.tif]

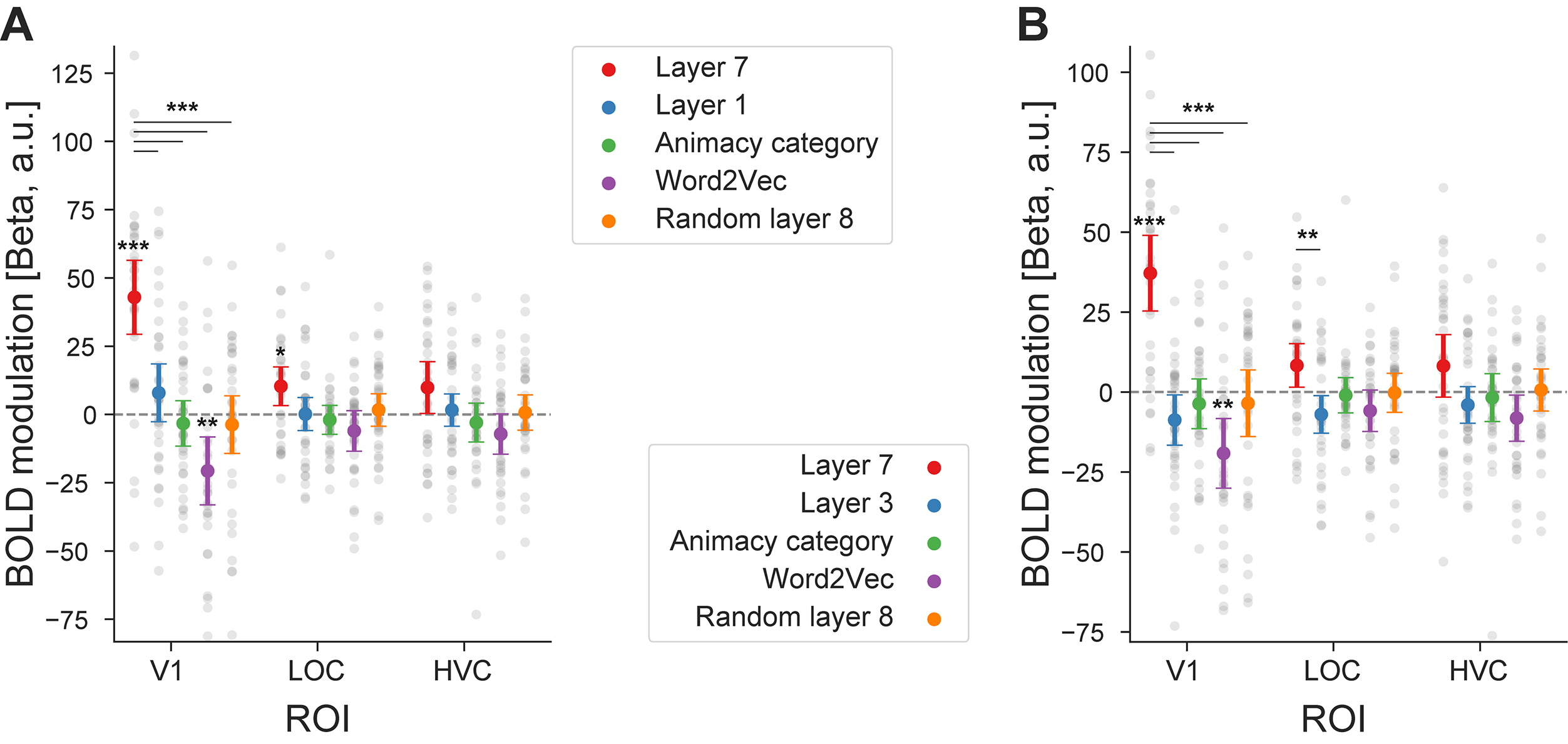

Supplement: S7 Fig — To ensure that our results were not specific to the DNN layers used in the primary analyses (see the Results section for details on the layer selection), we repeated the ROI analyses using surprise as indexed by the direct neighbors of our layers of interest. (A) ROI analysis in V1, LOC, and HVC using DNN layer 1 (blue) and layer 7 (red) as low-level and high-level surprise, respectively. (B) ROI analysis using layer 3 (blue) and layer 7 (red) surprise. Layer 9 was not explored, as it constitutes the class output layer of the DNN after softmax, and thus is categorical in nature. While compared to the primary analyses (Fig 6), using layer 2 and layer 8 surprise, quantitative differences are evident, qualitatively the results remain identical. That is, high-level surprise (here layer 7), best explains prediction error magnitudes compared to other surprise metric, particularly in V1. In contrast to layer 8 surprise, modulations by layer 7 were not quite as pronounced in later visual areas. Like layer 2, both layers 1 and 3 surprise failed to account for modulations of visual prediction errors. Error bars indicate the 95% within-subject confidence intervals. Gray dots denote individual subjects. P values are FDR corrected. *** p < 0.001, ** p < 0.01, * p < 0.05. Data and code that support these findings are available at: https://doi.org/10.34973/8e49-2012. (TIF) [file pbio.3002829.s007.tif]

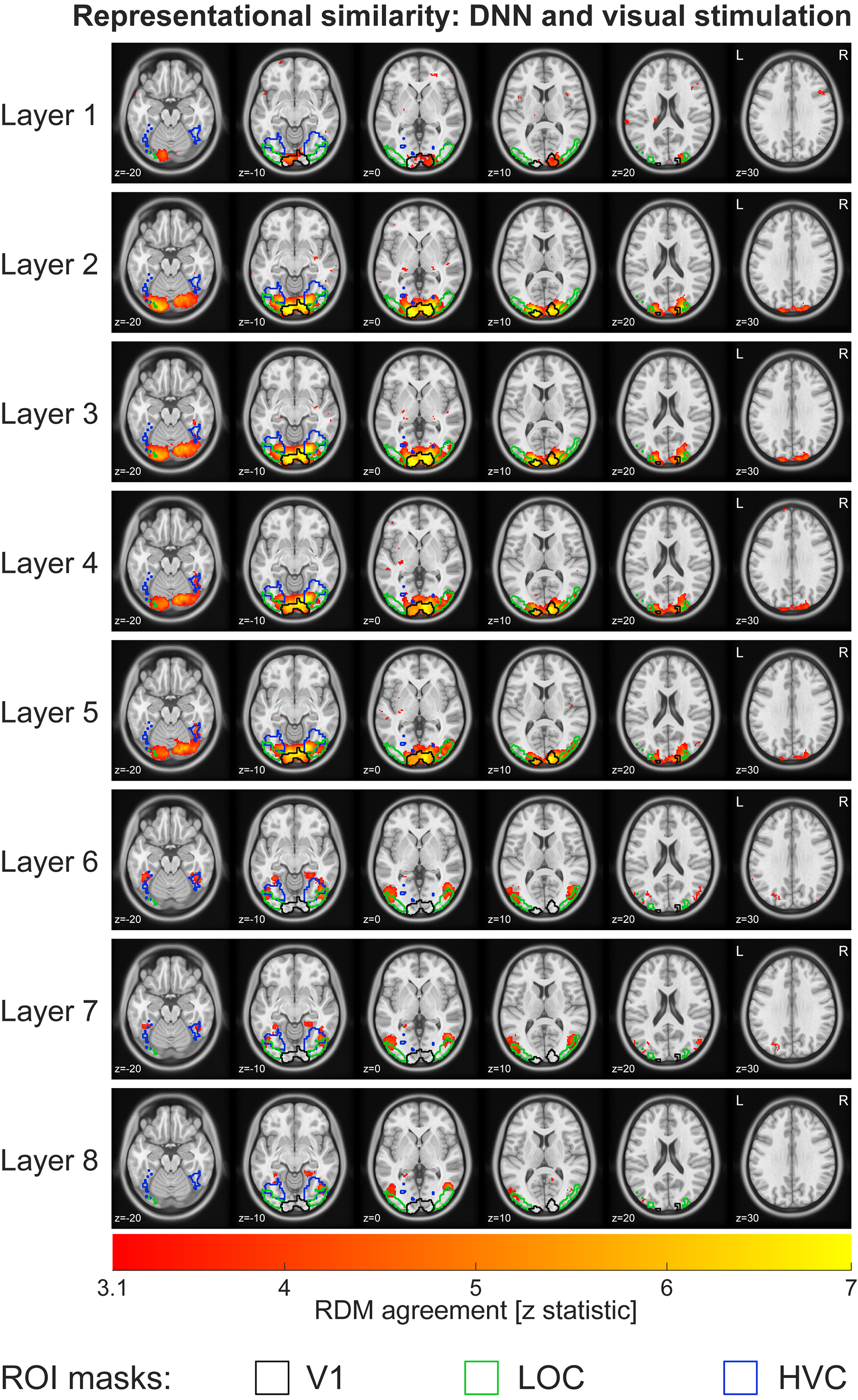

Supplement: S8 Fig — Early visual cortex (EVC) responses align more closely with early and intermediate DNN layers, indicative of low-level visual feature processing. Higher visual cortex (HVC) areas, like the fusiform gyrus, show a greater correlation with late DNN layers, representing high-level visual feature processing. Red to yellow cluster indicate statistically significant correlations between the layer RDMs (layers 1–8 separately for each panel), thresholded at z > 3.1 (i.e., p < 0.001, uncorrected). ROI masks are illustrated as colored outlines with black = V1, green = LOC, and blue = HVC. A strong contrast between earlier convolutional layers (1–5) and later dense layers (6–8) is evident. Particularly early layers strongly map onto EVC, while later layers map onto late ventral visual areas. We also note that while our ROI definition does separate the ventral visual stream roughly into 3 separate stages corresponding to early, intermediate, and high-level visual cortex, our HVC definition does not include major anterior HVC clusters particularly correlating with late DNN layer representations. Critically, our results do not depend on the specific ROI mask definition depicted, because larger ROI masks than those depicted here (see S5 Fig) and an alternative ROI mask definition using all stimulus-driven voxels (see S4 Fig) resulted in highly similar results as those obtained using the above depicted masks. Data and code that support these findings are available at: https://doi.org/10.34973/8e49-2012. (TIF) [file pbio.3002829.s008.tif]
