# Supplementary material for: High-level visual prediction errors in early visual cortex
Source: PLoS Biol. 2024 Nov 11;22(11):e3002829. doi: 10.1371/journal.pbio.3002829 (PMC11554119; doi:10.1371/journal.pbio.3002829)
Supplement: S1 Table — Listed are the contrasts of the parametric modulators (layer 8, layer 2, word category, animacy category, and random layer 8), as well as the contrast “unexpected minus expected stimuli” (Expectation suppression; S6 Fig) with corresponding area labels, numbers of voxels in the cluster, p value of the cluster, and peak z statistic. MNI coordinates indicate the X, Y, Z coordinates of the center of gravity for the cluster, as derived by FSL FEAT’s cluster function, in MNI space. Area labels are based on the center of gravity for the cluster and, especially for large clusters, additional areas encompassed by the cluster. (PDF) [file pbio.3002829.s009.pdf]

Contrast	Area labels	MNI coordinates			n voxel	*p* cluster	max *z*
		x	y	z			
High-level visual features (layer 8)	Occipital pole; Lateral Occipital Cortex, inferior division; Occipital Fusiform Gyrus	-19	-96	-1	779	4.9e-19	5.76
Low-level visual features (layer 2)	*No statistically significant clusters*						
Word category (word2vec)	Precuneous cortex (right)	19	-55	20	44	0.0384	-4.0
	Precuneous cortex (left)	-14	-63	24	44	0.0384	-4.2
Animacy category	*No statistically significant clusters*						
Random layer 8	Precuneous cortex	3	-66	30	48	0.0247	4.0
Expectation suppression (unexp. – exp.)	Superior parietal lobule; Lateral occipital cortex, superior division (left)	-36	-56	47	839	9.9e-20	4.5
	Middle and inferior frontal gyrus; Precentral gyrus (left)	-44	6	33	325	4.0e-10	4.8
	Paracingulate gyrus	1	13	48	265	9.8e-9	4.9
	Superior parietal lobule; Lateral occipital cortex, superior division (right)	31	-67	37	232	6.0e-8	5.3
	Frontal operculum cortex; Anterior insula (left)	-37	18	3	153	8.1e-6	4.4
	Inferior temporal gyrus; Lateral occipital cortex, inferior division	-45	-58	-13	150	9.8e-6	5.0
	Frontal operculum cortex; Anterior insula (right)	39	22	2	136	2.5e-5	4.2
	Middle and inferior frontal gyrus (right)	48	29	24	110	1.0e-4	4.6
	Precentral gyrus (right)	42	7	32	96	1.0e-4	4.2
	Middle and inferior frontal gyrus (left)	-41	33	18	53	0.0175	4.2

S1 Table. Brain areas showing significant modulations of BOLD responses (GRF cluster corrected). Listed are the contrasts of the parametric modulators (layer 8, layer 2, word category, animacy category, and random layer 8), as well as the contrast 'unexpected minus expected stimuli' (Expectation suppression; S6 Fig) with corresponding area labels, numbers of voxels in the cluster, p value of the cluster, and peak z statistic. MNI coordinates indicate the X, Y, Z coordinates of the centre of gravity for the cluster, as derived by FSL FEAT's cluster function, in MNI space. Area labels are based on the centre of gravity for the cluster and, especially for large clusters, additional areas encompassed by the cluster.
