# Supplementary material for: High-level visual prediction errors in early visual cortex
Source: PLoS Biol. 2024 Nov 11;22(11):e3002829. doi: 10.1371/journal.pbio.3002829 (PMC11554119; doi:10.1371/journal.pbio.3002829)
Supplement: S2 Table — VIFs were computed on the first-level (i.e., run-level) fMRI design matrices, as supplied to FSL FEAT, and subsequently averaged across runs and participants. Overall, obtained VIFs are low—commonly VIFs >5 are considered problematic. Additionally, the VIF for the low-level visual surprise regressor is lower (i.e., better) compared to high-level visual surprise, suggesting that the absence of a modulation of neural responses by low-level surprise is unlikely to be caused by problems with variance partitioning due to collinearity of the predictors in the GLM. (PDF) [file pbio.3002829.s010.pdf]

Regressor	Variance inflation factor
Expected	2.18
Unexpected	2.19
No Go	1.55
High-level visual surprise (layer 8)	1.88
Low-level visual surprise (layer 2)	1.42
Response category (animacy)	1.44
Word category surprise (word2vec)	1.50
Untrained layer 8 (random DNN layer 8)	1.22

S2 Table. Variance inflation factor (VIF) for each regressor. VIFs were computed on the first-level (i.e., run-level) fMRI design matrices, as supplied to FSL FEAT, and subsequently averaged across runs and participants. Overall, obtained VIFs are low – commonly VIFs >5 are considered problematic. Additionally, the VIF for the low-level visual surprise regressor is lower (i.e., better) compared to high-level visual surprise, suggesting that the absence of a modulation of neural responses by low-level surprise is unlikely to be caused by problems with variance partitioning due to collinearity of the predictors in the GLM.
