# Supplementary material for: High-level visual prediction errors in early visual cortex
Source: PLoS Biol. 2024 Nov 11;22(11):e3002829. doi: 10.1371/journal.pbio.3002829 (PMC11554119; doi:10.1371/journal.pbio.3002829)
Supplement: S3 Table — We found reliable modulations by the high-level visual model (layer 8) throughout all 3 ROIs, encompassing early (V1) and high level (HVC). P values are FDR corrected. (PDF) [file pbio.3002829.s011.pdf]

ROI	Contrast	Test statistic	P value	Effect size
V1	Layer 8	$t_{(32)} = 6.79$	$p < 0.001$	$d_z = 1.18$
V1	Layer 2	$W = 190$	$p = 0.199$	$r = -0.32$
V1	Animacy category	$t_{(32)} = -2.32$	$p = 0.068$	$d_z = -0.40$
V1	Word2Vec	$W = 112$	$p = 0.020$	$r = -0.60$
V1	Random Layer 8	$W = 253$	$p = 0.719$	$r = -0.10$
LOC	Layer 8	$W = 126$	$p = 0.029$	$r = 0.55$
LOC	Layer 2	$t_{(32)} = -0.68$	$p = 0.630$	$d_z = -0.12$
LOC	Animacy category	$W = 137$	$p = 0.039$	$r = -0.51$
LOC	Word2Vec	$t_{(32)} = -1.37$	$p = 0.298$	$d_z = -0.24$
LOC	Random Layer 8	$t_{(32)} = 0.21$	$p = 0.892$	$d_z = 0.04$
HVC	Layer 8	$t_{(32)} = 2.59$	$p = 0.043$	$d_z = 0.45$
HVC	Layer 2	$t_{(32)} = -0.70$	$p = 0.666$	$d_z = -0.12$
HVC	Animacy category	$W = 232$	$p = 0.579$	$r = -0.17$
HVC	Word2Vec	$t_{(32)} = -2.27$	$p = 0.065$	$d_z = -0.39$
HVC	Random Layer 8	$t_{(32)} = 0.19$	$p = 0.852$	$d_z = 0.03$

S3 Table. Results of one sample t-tests and Wilcoxon signed rank test contrasting parameter estimates of the parametric modulators against zero (no modulation). We found reliable modulations by the high-level visual model (Layer 8) throughout all three ROIs, encompassing early (V1) and high level (HVC). P values are FDR corrected.
