# Supplementary material for: High-level visual prediction errors in early visual cortex
Source: PLoS Biol. 2024 Nov 11;22(11):e3002829. doi: 10.1371/journal.pbio.3002829 (PMC11554119; doi:10.1371/journal.pbio.3002829)
Supplement: S4 Table — The high-level visual model (layer 8) modulated sensory responses significantly more than any of the 4 control models in V1, as well as some models in LOC and HVC. P values are FDR corrected. (PDF) [file pbio.3002829.s012.pdf]

ROI	Contrast	Test statistic	P value	Effect size
V1	Layer 8 vs Layer 2	$t_{(32)} = 8.05$	$p < 0.001$	$d_z = 1.40$
V1	Layer 8 vs Animacy category	$t_{(32)} = 5.71$	$p < 0.001$	$d_z = 0.99$
V1	Layer 8 vs Word2Vec	$t_{(32)} = 5.91$	$p < 0.001$	$d_z = 1.03$
V1	Layer 8 vs Random layer 8	$t_{(32)} = 6.66$	$p < 0.001$	$d_z = 1.16$
V1	Layer 2 vs Animacy category	$t_{(32)} = 0.44$	$p = 0.737$	$d_z = 0.08$
V1	Layer 2 vs Word2Vec	$W = 218.0$	$p = 0.440$	$r = 0.22$
V1	Layer 2 vs Random layer 8	$W = 216.0$	$p = 0.440$	$r = -0.23$
V1	Animacy category vs Word2Vec	$t_{(32)} = 0.28$	$p = 0.810$	$d_z = 0.05$
V1	Animacy category vs Random layer 8	$t_{(32)} = -0.66$	$p = 0.645$	$d_z = -0.11$
V1	Word2Vec vs Random layer 8	$W = 204.0$	$p = 0.322$	$r = -0.27$
LOC	Layer 8 vs Layer 2	$t_{(32)} = 4.24$	$p = 0.001$	$d_z = 0.74$
LOC	Layer 8 vs Animacy category	$W = 108.0$	$p = 0.010$	$r = 0.61$
LOC	Layer 8 vs Word2Vec	$W = 148.0$	$p = 0.060$	$r = 0.47$
LOC	Layer 8 vs Random layer 8	$W = 158.0$	$p = 0.078$	$r = 0.44$
LOC	Layer 2 vs Animacy category	$t_{(32)} = 0.71$	$p = 0.631$	$d_z = 0.12$
LOC	Layer 2 vs Word2Vec	$W = 261.0$	$p = 0.780$	$r = -0.07$
LOC	Layer 2 vs Random layer 8	$t_{(32)} = -0.64$	$p = 0.607$	$d_z = -0.11$
LOC	Animacy category vs Word2Vec	$W = 238.0$	$p = 0.640$	$r = -0.15$
LOC	Animacy category vs Random layer 8	$t_{(32)} = -1.63$	$p = 0.261$	$d_z = -0.28$
LOC	Word2Vec vs Random layer 8	$t_{(32)} = -1.0$	$p = 0.490$	$d_z = -0.17$
HVC	Layer 8 vs Layer 2	$t_{(32)} = 2.85$	$p = 0.028$	$d_z = 0.50$
HVC	Layer 8 vs Animacy category	$t_{(32)} = 2.32$	$p = 0.081$	$d_z = 0.40$
HVC	Layer 8 vs Word2Vec	$t_{(32)} = 3.13$	$p = 0.016$	$d_z = 0.54$
HVC	Layer 8 vs Random layer 8	$t_{(32)} = 1.84$	$p = 0.189$	$d_z = 0.32$
HVC	Layer 2 vs Animacy category	$t_{(32)} = 0.13$	$p = 0.900$	$d_z = 0.02$
HVC	Layer 2 vs Word2Vec	$t_{(32)} = 1.03$	$p = 0.488$	$d_z = 0.18$
HVC	Layer 2 vs Random layer 8	$t_{(32)} = -0.65$	$p = 0.628$	$d_z = -0.11$
HVC	Animacy category vs Word2Vec	$W = 199.0$	$p = 0.291$	$r = 0.29$
HVC	Animacy category vs Random layer 8	$t_{(32)} = -0.77$	$p = 0.612$	$d_z = -0.13$
HVC	Word2Vec vs Random layer 8	$t_{(32)} = -1.61$	$p = 0.252$	$d_z = -0.28$

S4 Table. Results of paired t-tests and Wilcoxon signed rank test, contrasting the parameter estimates of the parametric modulators in a pair-wise fashion within each ROI. The high-level visual model (layer 8) modulated sensory responses significantly more than any of the four control models in V1, as well as some models in LOC and HVC. P values are FDR corrected.
