# Supplementary material for: High-level visual prediction errors in early visual cortex
Source: PLoS Biol. 2024 Nov 11;22(11):e3002829. doi: 10.1371/journal.pbio.3002829 (PMC11554119; doi:10.1371/journal.pbio.3002829)
Supplement: S5 Table — For each participant and within each ROI, we extracted the single-trial parameter estimates (BOLD) from the localizer runs. On this data, we trained an 8-class classifier, with the 8 classes corresponding to the 8 objects seen by the participant. Next, we tested the classifier on the single-trial parameter estimates from the main task runs. To improve SNR, we averaged the single-trial estimates for each object and expectation condition (expected or unexpected) within runs. Finally, we averaged the obtained decoding accuracies across runs to obtain a mean decoding accuracy for expected and unexpected trials per participant. On average decoding of the object stimuli was well above chance (12.5%), but did not reliably differ between expected and unexpected stimuli in any of the 3 ROIs (V1: t(32) = −0.51, p = 0.613; LOC: t(32) = −1.02, p = 0.315; HVC: t(32) = 0.55, p = 0.588). (PDF) [file pbio.3002829.s013.pdf]

ROI	Expectation status	Decoding accuracy [mean ± 95% CI]
V1	Expected	73.1% ± 3.3%
	Unexpected	73.8% ± 3.3%
LOC	Expected	63.1% ± 2.2%
	Unexpected	64.5% ± 2.6%
HVC	Expected	47.5% ± 3.0%
	Unexpected	46.9% ± 3.0%

S5 Table. Decoding of stimulus identity across expectation conditions. For each participant and within each ROI we extracted the single-trial parameter estimates (BOLD) from the localizer runs. On this data we trained an 8-class classifier, with the 8 classes corresponding to the 8 objects seen by the participant. Next, we tested the classifier on the single-trial parameter estimates from the main task runs. To improve SNR, we averaged the single-trial estimates for each object and expectation condition (expected or unexpected) within runs. Finally, we averaged the obtained decoding accuracies across runs to obtain a mean decoding accuracy for expected and unexpected trials per participant. On average decoding of the object stimuli was well above chance (12.5%), but did not reliably differ between expected and unexpected stimuli in any of the three ROIs (V1: $t_{(32)} = -0.51$, $p = 0.613$; LOC: $t_{(32)} = -1.02$, $p = 0.315$; HVC: $t_{(32)} = 0.55$, $p = 0.588$).
