# Supplementary material for: High-level visual prediction errors in early visual cortex
Source: PLoS Biol. 2024 Nov 11;22(11):e3002829. doi: 10.1371/journal.pbio.3002829 (PMC11554119; doi:10.1371/journal.pbio.3002829)
Supplement: Attachment — Submitted filename: plos_REPLY_TO_REVIEWERS_rev1_v5.pdf [file pbio.3002829.s014.pdf]

We thank the reviewers for their helpful comments. Below we provide a point-by-point reply to the reviewer comments. Our replies are marked in blue. Changes and additions in the manuscript are marked in blue as well.

Reviewer #1:

The study is statistically well powered, the analytical approach ingenious, and the results seem mostly robust. The manuscript thus
constitutes and important addition to the literature on predictive processing by showing that results previously suggested by electrophysiology in non-human primates apply also to human visual processing and extend this literature by showing that these principles extend beyond the brain areas and stimuli tested in these studies.

We thank the reviewer for the positive evaluation of our study.

1. The finding that early layer representational spaces explain little to no variance during prediction errors is somewhat surprising. Although the finding is consistent with the overall hypothesis, I was wondering whether it is possible that the layer RDMs are highly correlated with each other, thus limiting the ability to partition their respective associated variance? Although the authors report that their image selection procedure minimized such correlations, the actual result correlations are not reported. It would be useful to provide evidence that the null results is not due to a problem with the predictors in the GLM. Variance inflation factors could be one useful criterion to determine whether there is an issue or not.

Our choice to focus our primary analyses on two distant layers of the DNN was indeed motivated by the potential problem of high correlations between (especially neighboring) DNN layers. We agree that ruling out that our null results arose due to problems with the predictors in the GLM is nonetheless important for the interpretation of our results. Therefore, we followed the reviewer's suggestion and computed variance inflation factors (VIF) for our predictors. We now report the full set VIFs in the supplemental material (p. 54):

**Regressor**	**Variance inflation factor**
Expected	2.18
Unexpected	2.19
No Go	1.55
High-level visual surprise (layer 8)	1.88
Low-level visual surprise (layer 2)	1.42
Response category (animacy)	1.44
Word category surprise (word2vec)	1.50
Untrained layer 8 (random DNN layer 8)	1.22

Supplementary Table 2. Variance inflation factor (VIF) for each regressor. VIFs were computed on the first-level (i.e., run-level) fMRI design matrices, as supplied to FSL FEAT, and subsequently averaged across runs and participants. Overall, obtained VIFs are low – commonly VIFs >5 are considered problematic. Additionally, the VIF for the low-level visual surprise regressor is lower (i.e., better) compared to high-level visual surprise, suggesting that the absence of a modulation of neural responses by low-level surprise is unlikely to be caused by problems with variance partitioning due to collinearity of the predictors in the GLM.

We also highlight the most important VIF results and associated conclusion in the Results section: "Additionally, variance inflation factors (VIF; Supplementary Table 2) were smaller for layer 2 (VIF =

1.42) than layer 8 surprise (VIF = 1.88), suggesting that the absence of a significant modulation by low-level surprise was not due to problems with variance partitioning due to collinearity of the predictors in the GLM. In fact, all VIFs were significantly lower than the suggested threshold of VIF < 5 (34).” (line 328).

2. The authors report that they used a decoding analysis to further refine the voxels within the otherwise anatomically delineated regions of interest. This decoding analysis was done on the basis of the localizer data. Several questions arise:
1. Was the same voxel selection procedure used to analyze the representational geometry of the localizer data? If so, the procedure seems potentially problematic because it might bias results towards finding the "feedforward" tuning properties; if not, comparisons between the prediction error and the localizer data may be invalid because of the different selection criteria.

We thank the reviewer for bringing up this important point.
To briefly summarize the procedure: For each participant we refined one mask per anatomically defined ROI using the localizer data. We then applied these masks to the main task (i.e., independent data). No ROI analysis was performed on the localizer data itself. The analysis of the representational geometry of the localizer data was exclusively performed as a whole brain analysis (Fig. 3). We believe that constraining the anatomically defined masks is beneficial, as large parts of each anatomically defined ROI were not activated by the presented stimuli, given broad anatomical labels (e.g. EVC anatomical masks were often >4,000 voxels for a given participant), small stimulus size (6 DVA), and the receptive fields in each ROIs (particularly in EVC).
We agree with the reviewer that the specific ROI selection procedure could in principle have biased results towards feedforward tuning. However, the critical results, that EVC responses scaled with high-level and not low-level surprise, appear opposite to what we would expect should our mask selection procedure indeed have biased the results towards feedforward tuning.
Furthermore, we now sought to empirically address the issue of the specific ROI mask voxel selection procedure, by reanalyzing our data using more liberal ROI masks that were only constrained to stimulus responsive voxels (localizer run: z > 3.1 for the contrast stimulus > baseline) within the three anatomically defined ROIs. Thus, these ROI masks only ensure that voxels considered in the analysis are stimulus responsive and within the anatomical ROIs, resulting in large masks of on average >1,200 voxels per mask, but did not require any specific representational geometry. Results of this ROI analysis as well as the associated ROI masks are depicted in Supplementary Fig. 4 (p. 47-48). Overall results were highly similar to those reported in the manuscript (Fig. 6), and in fact show more robust modulations of BOLD responses by high-level (layer 8) surprise particularly in LOC and HVC. However, given that our ROI mask choice was defined a priori, using an identical procedure as our previous studies (e.g. Richter et al., 2018), we prefer to retain the original masks and report this control analysis in the supplemental material, even though the results are in fact more favourable for our conclusions in terms of statistical significance.

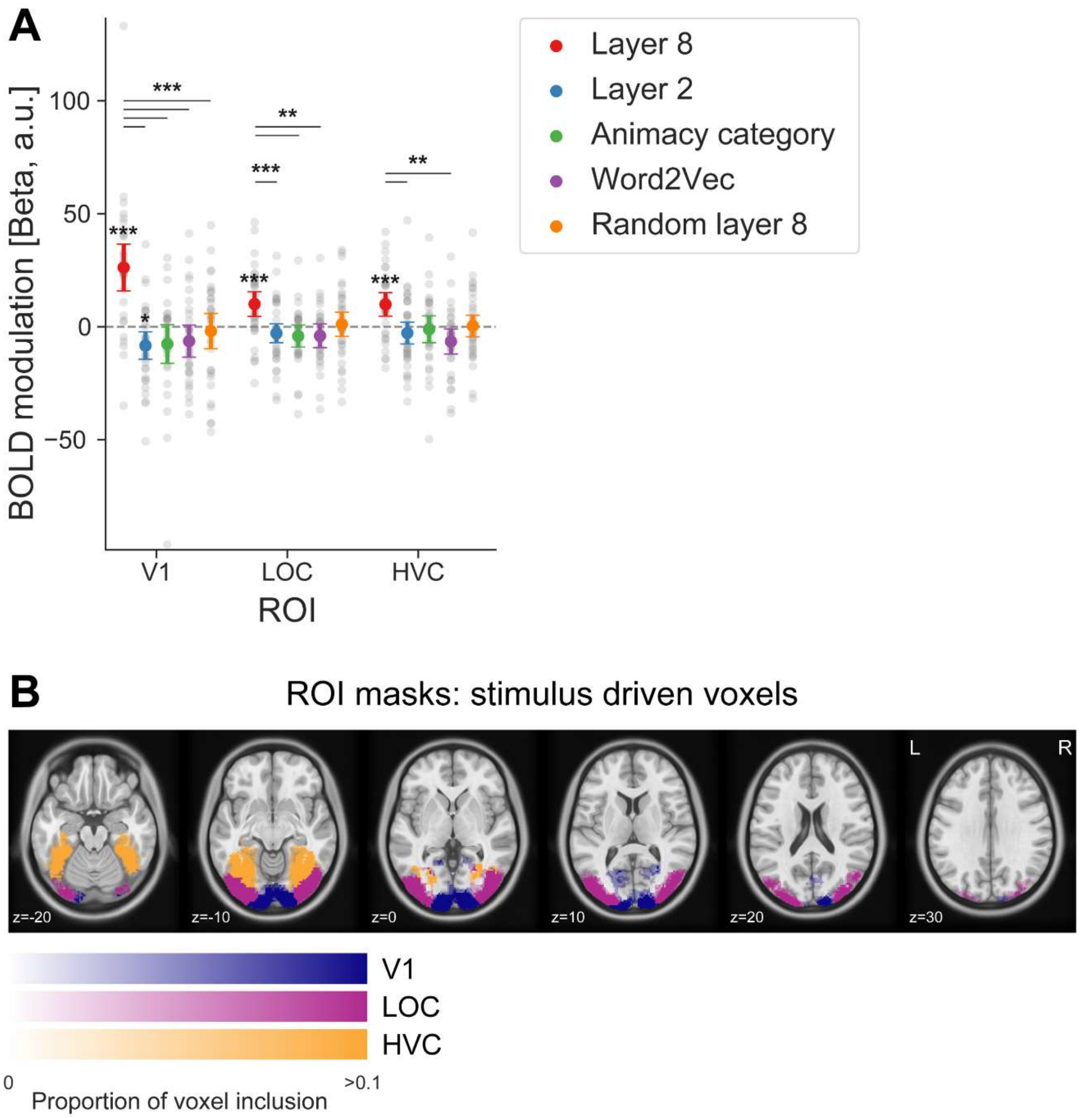

Supplementary Figure 4. Visual responses scale with high-level surprise across the visual hierarchy in stimulus-driven voxels. A) Control ROI analysis using all stimulus driven within the anatomically defined V1, LOC and HVC masks. For ROI mask creation, stimulus driven was defined as a significant activation ($z > 3.1$) of voxels by the presentation of the object images during the independent localizer run. Thus, compared to the ROI masks used in the main analyses, these ROI masks are larger, encompassing all visually driven voxels within the ROIs. Average mask size in voxels: V1 = 1254, LOC = 2443, HVC = 1383. Results show that prediction error magnitudes are best explained by high-level visual feature dissimilarity across all three ROIs. Error bars indicate the 95% within-subject confidence intervals. Gray dots denote individual subjects. P values are FDR corrected. *** $p < 0.001$, ** $p < 0.01$. **B)** Voxels included in the ROI masks using all stimulus-driven voxels within the anatomically defined masks. Color indicates the ROI: Blue = V1, Purple = LOC, Orange = HVC. Notably, the resulting masks are significantly larger and extend further anterior in HVC compared to the original decoding-based masks. Opacity indicates the proportion of participants whose individual masks included

the voxel. For visualization, full opacity corresponds to a proportion of 0.1, with voxel inclusion proportions reaching up to 1.

We now also briefly refer to these results in the Results section:

“Finally, to ensure that our results were not dependent on the exact ROI mask definition and mask sizes, we repeated the analysis for an alternative ROI mask definition (using all stimulus driven voxels within the anatomically defined masks) and across multiple mask sizes. Results were qualitatively identical across ROI definitions (Supplementary Figure 4) and mask sizes (Supplementary Figure 5).” (line 361)

2. When the ROI sizes were extended to account for the possibly confounding effects of voxel number, was this restricted to voxels that contributed to the decoding?

Voxel selection was not explicitly restricted to voxels that significantly contribute to decoding, however when extending mask sizes for the control analysis, we first added voxels with the next highest decoding accuracy. Thereby even the largest masks (500 voxels) contained voxels which on average contributed to reliable decoding of object identity during the localizer (decoding accuracy of 500 voxel masks: EVC = 78.7%; LOC = 52.3%; HVC = 41.4%; chance = 12.5%). Thus, while not explicit set as a criterion (almost all) voxels significantly contributed to object decoding.

3. Did the representational geometry of the non-selected voxels in the respective ROIs resemble any of the DNN layers in the prediction error condition? This would give an indication as to whether the selectivity of neural populations factors into the results the authors report.

As noted above in our reply to question 2.1., masks encompassing non-selected voxels will contain many voxels which did not respond to the stimuli. For example, the average anatomical EVC mask was >4000 voxels, with only ~1200 of these on average being stimulus responsive. Thereby performing an analysis using non-selected voxels will contain ~2800 voxels that are not stimulus driven. Hence it is unlikely that these voxels scale reliably with any surprise metric. To empirically verify, we performed the ROI analysis using masks containing voxels which were not selected for the 500 voxel ROI masks.

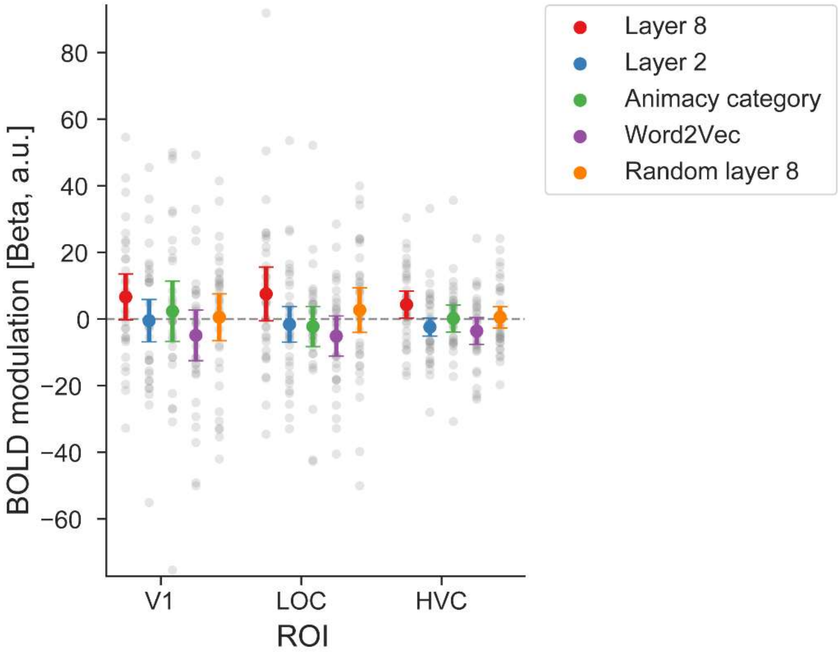

Figure, ROI analysis using non-selected voxels.

In fact, the analysis rationale and its results are similar to a control analysis we performed and reported in the previous version of the manuscript as Supplementary Figure 1 (now Supplementary Figure 2; p. 45).

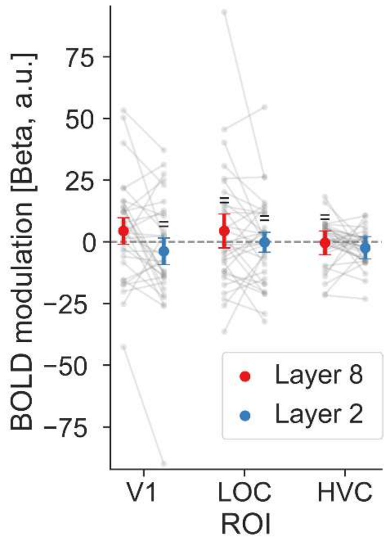

Supplementary Figure 2. No modulation of neural responses by visual surprise in stimulus uninformative voxels. Control ROI analysis using stimulus uninformative voxels in early visual (V1), intermediate (LOC) and higher visual cortex (HVC; encompassing occipito-temporal sulcus and fusiform cortex). ROI masks were defined using the same procedure as outlined in the *Region of interest (ROI) analysis* paragraph of the *Materials and Methods* sections, except for that voxel selection was performed by choosing the least informative (i.e., lowest decoding performance) voxels for object identity decoding during the localizer runs. No modulation of neural responses by high-level (layer 8) or low-level (layer 2) surprise was observed in any ROI containing voxels uninformative about the stimuli. These results further suggest that the modulation of visual responses by high-level surprise (Figure 5) is specific to stimulus-selective voxels and not an unspecific global surprise signal. Error bars indicate the 95% within-subject confidence intervals. Gray dots denote individual subjects. = $BF_{10} < 1/3$.

We think that showing that stimulus uninformative voxels do not scale reliably with surprise is an important addition to the manuscript, as these results demonstrate that modulations by surprise are not an unspecific and global modulation affecting all neural populations in visual cortex.
The objective noted by the reviewer was to explore how tuning might affect surprise modulations. While we believe that further and more detailed exploration of potential interactions between feedforward tuning and surprise scaling would be an interesting next step, it appeared beyond the scope of the present manuscript. Indeed, more precise RF mapping and other ROI localization techniques might be important to further explore this question. We believe that our whole-brain analyses (Fig. 5) do already provide some intuition for whether and how scaling of prediction errors may change across the visual hierarchy. As evident in those results (copied below for convenience) there is some indication that high-level surprise other than layer 8 contributes to the prediction error in medial parts of EVC and beyond (see: orange colours in Fig. 5 corresponding to layer 6-7), as well as some areas showing scaling by low to intermediate surprise (see: light blue to light green colours in Fig. 5 corresponding to layer 3-4).

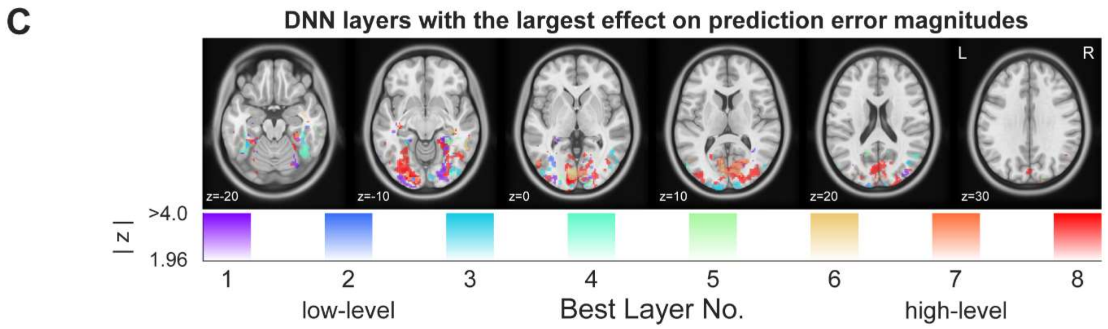

3. Is it possible that the predictor letters drive the results, i.e., could the authors provide a control analysis in which they determine similarity between the predictor letters and apply that to the image-evoked activity?

We agree that it is critical to ascertain that the predictor letters did not drive the results. Moreover, we agree that determining the similarity between predictor letters and stimuli is one approach. However, we argue that this concern is effectively ruled out by our experimental design. We now better clarify this critical aspect of our design in the manuscript's Materials and Methods section after explaining the stimulus selection:

> "From the remaining 213 stimuli eight images were selected for each participant, including four animate and four inanimate images. [...] Images were randomly paired with a specific letter for each participant. Thus, most participants saw different images and in the case of the same image being used for more than one participant, the individual letter-image pairings were different as well. We made this choice to rule out, by design, that specific images or cue-image pairs drive the results, for example due to high representational similarity for some cue-image pairs. Moreover, because participants were shown different stimuli (eight selected from a set of 213 stimuli), our results are unlikely to be contingent on any specific set of eight stimuli. Similarly, because the pairing of the letter cues and image stimuli was randomized between participants, even participants who saw the same stimuli did not learn the same letter-image pairs (approximately 3.7x10^{21} possible combinations), thereby effectively ruling out that specific cue-image combinations account for the results." (line 621)

While this does not formally rule out any particular cue-image pairs contributing more to the overall results, we believe that the vast number of combinations (approximately 3.7x10^{21}) and sample size (n=33) makes significant individual cue-image contributions to drive the results exceedingly unlikely.

4. It is often recommended to z-transform correlation coefficients before subjecting them to t-tests. I suggest the authors try that here, too.

Agreed, we corrected this oversight and updated the analyses and manuscript accordingly – results remain qualitatively unchanged.

> "Finally, we z transformed the resulting correlation coefficients and subjected them to one-sample t-tests across subjects for each voxel". (line 911)

Minor:

- The methods section has several formulations that sound a bit weird in terms of language, e.g., in line 634 ("boilerplate"), 637 and 653 ("images were found"), 722 (presumably CSF and white matter time courses).

Thank you for these suggestions. Indeed, some formulations in the methods may seem unusual. The specific method text was generated by fMRIprep and requested by the authors (Esteban et al., 2019) was copied into the methods. We clarify this in line 712:

> "MRI preprocessing pipeline description generated by fMRIprep".

- The reference to Behzadi et al. (line 677) seems to be missing.
We thank the reviewer for the attention to detail and added the missing reference.

Reviewer #2 (Arjen Alink):
The topic and the conclusions drawn by this study are of sufficient general interest to be considered for publication in PLOS Biology. In its present form, however, the findings reported do not sufficiently support the main conclusions drawn by the authors, as pointed out in the main issues listed below. Resolving these issues would require additional analyses and/or a substantial 'toning-down' of the conclusions made. The latter could significantly affect the impact of the paper, and its suitability for PLOS Biology.

We thank the reviewer for the overall positive evaluation of our study, as well as the critical and constructive feedback.

Major issue 1
The authors assume that layer 2 and layer 8 of their trained DNN represent low and high-level image features respectively. They, however, do not provide a compelling argument for this assumption, which is crucial to the main findings of this study. Based on Figure 3, it is impossible to say which layer best explains RDMs in EVC, LOC and HVC, as these areas are not highlighted. The authors should instead, or in addition, show how well each layer explains RDM characteristics for the EVC, LOC and HVC ROIs (using all voxels). If, e.g., layers 1 and 3 also robustly explain EVC RDMs, they need to be considered as well. Currently, the results presented (e.g., in 5a) look very convincing. However, it is not clear whether this is a result of the choice of layers. Therefore, I would strongly insist on the authors reporting their main result (Figure 5B and 5D) for all layers, at a minimum via a supplementary figure.

The reviewer touches on several important points. First, paraphrasing the reviewer, the key problems are: (1) what do layer 2 and 8 of the DNN represent, (2) how do ROIs map onto Figure 3, and (3) are the results specific to the two analyzed DNN layers.

First, we agree that understanding what kind of features layer 2 and layer 8 represent is important. Before turning to this question in more detail, we believe that there is already evidence to support that layer 2 reflects low-level and layer 8 high-level features. The convergence of DNN and visual cortical representations suggests a gradient from low- to high-level features (Fig. 3), like the visual cortical gradient, supporting that early layers of the DNN are tuned to low-level and later layers to high-level features. Next, prior work using similar visual DNNs has explored this question in more detail and provided evidence to support this assumption, again by showing a convergence of visual cortical and DNN layer hierarchies (Guclu & van Gerven, 2015; Mehrer et al., 2021; Yamins et al., 2014). That said, we agree with the reviewer that additional, more direct evidence is warranted to support the notion that layer 2 of the specific DNN used here reflects (largely) low-level visual and layer 8 high-level features. To this end we now visualize the features layer 2 and 8 of the DNN are tuned to; for details, please refer to our reply to Major issue 2, which directly relates to this question.

A second aspect the reviewer correctly highlights is the shortcoming of our previous figures to adequately illustrate which cortical areas the utilized ROI masks contain and how they relate to the results reported in Fig. 3. Indeed, a similar concern was voiced by R3, comment 6. Following these suggestions and to help readers better understand the location and extend of our ROI masks, and

thereby relate the ROI masks Fig. 3, we now illustrate the ROI masks in a new panel added to Fig. 3B (p. 7), which we refer to in the associated Results section:

> "Our whole-brain results were corroborated by an ROI analysis, depicted in Figure 5B (ROI masks are illustrated in Figure 3B)." (line 232).

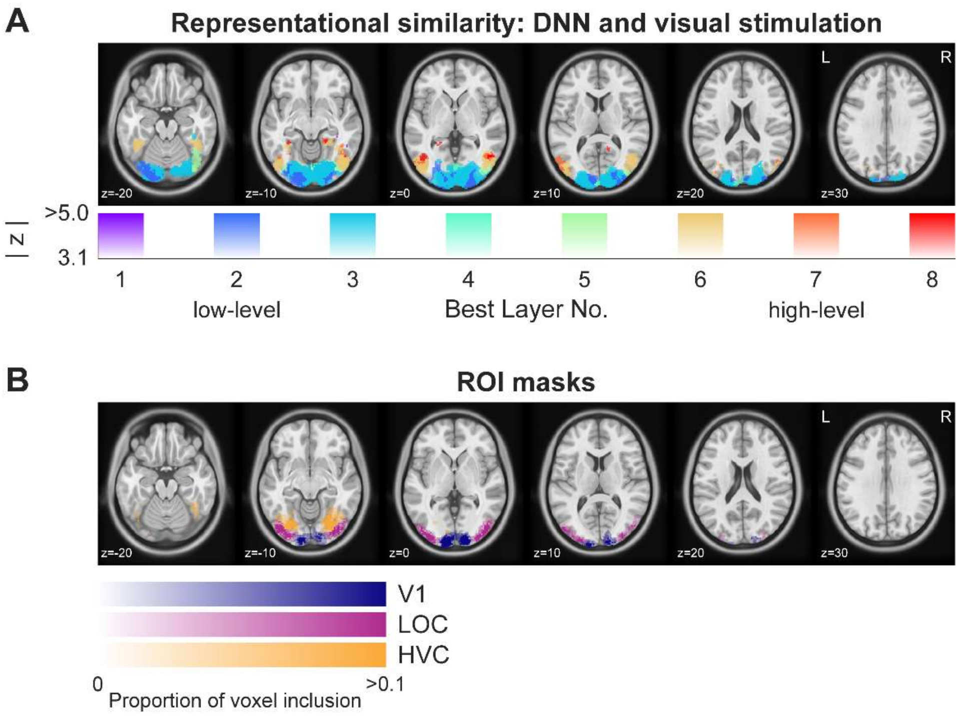

Figure 3. Convergence of DNN and visual cortical representations showing a gradient from low- to high-level features in prediction-free contexts. A) Representational similarity analysis of visual responses shows that (feedforward) visual responses during the prediction-free localizer were best explained by a gradient of low-level to high-level visual features going up the ventral visual hierarchy. Early visual cortex (EVC) responses aligned more closely with early DNN layers, indicative of low-level visual feature processing (depicted in cold colours: purple to blue). Higher visual cortex (HVC) areas, like the fusiform gyrus, show a greater correlation with late DNN layers, representing high-level visual feature processing (illustrated in warm colours: yellow to red). Analysis was masked to visual cortex and thresholded at z > 3.1 ($p < 0.001$, uncorrected) of the RSA. **B)** ROI masks, depicting voxels included in the anatomically and functionally defined masks. Color indicates the ROI: Blue = V1, Purple = LOC, Orange = HVC. Opacity indicates the proportion of participants whose individual masks included the voxel. For visualization, full opacity corresponds to a proportion of 0.1, with voxel inclusion proportions reaching up to ~0.7.

The masks well separate visual cortex into three distinct clusters, roughly corresponding to early, intermediate and high-level visual cortex. However, we do acknowledge that our HVC mask (orange in Fig. 3B) is more posterior and medial than the clusters of visual cortices best explained by high-level (layer 8) representations during the prediction-free localizer (see red in Fig. 3A). Thus, our HVC mask did not contain the "highest" level visually responsive areas at the terminus of the ventral visual stream. While it is interesting to further explore prediction error scaling dependent on feedforward tuning, we believe that this potential shortcoming of our HVC mask does not impede our primary conclusions. In fact, in reply to a comment by R1 (comment 2.1.) we performed an additional control ROI analysis, using all stimulus-driven voxels within the three anatomically defined ROIs, thus resulting in significantly broader ROI masks than used in our primary ROI analyses.

Results in these less constrained masks were highly similar, and in fact more robust, than those we report in our main analyses. For convenience we copied the relevant figure below, which we now include as Supplementary Figure 4 (p. 47) in the manuscript. Combined, the available data suggests that differences in ROI definition do not significantly impact our conclusions. Thus, we believe that, while exploring prediction error modulations across the hierarchy in a more fine-grained ROI fashion and contingent on feedforward tuning is an interesting extension of the present work, our ROI and whole brain analyses support the conclusions that visual responses across the visual hierarchy primarily scale with high-level, but not low-level surprise.

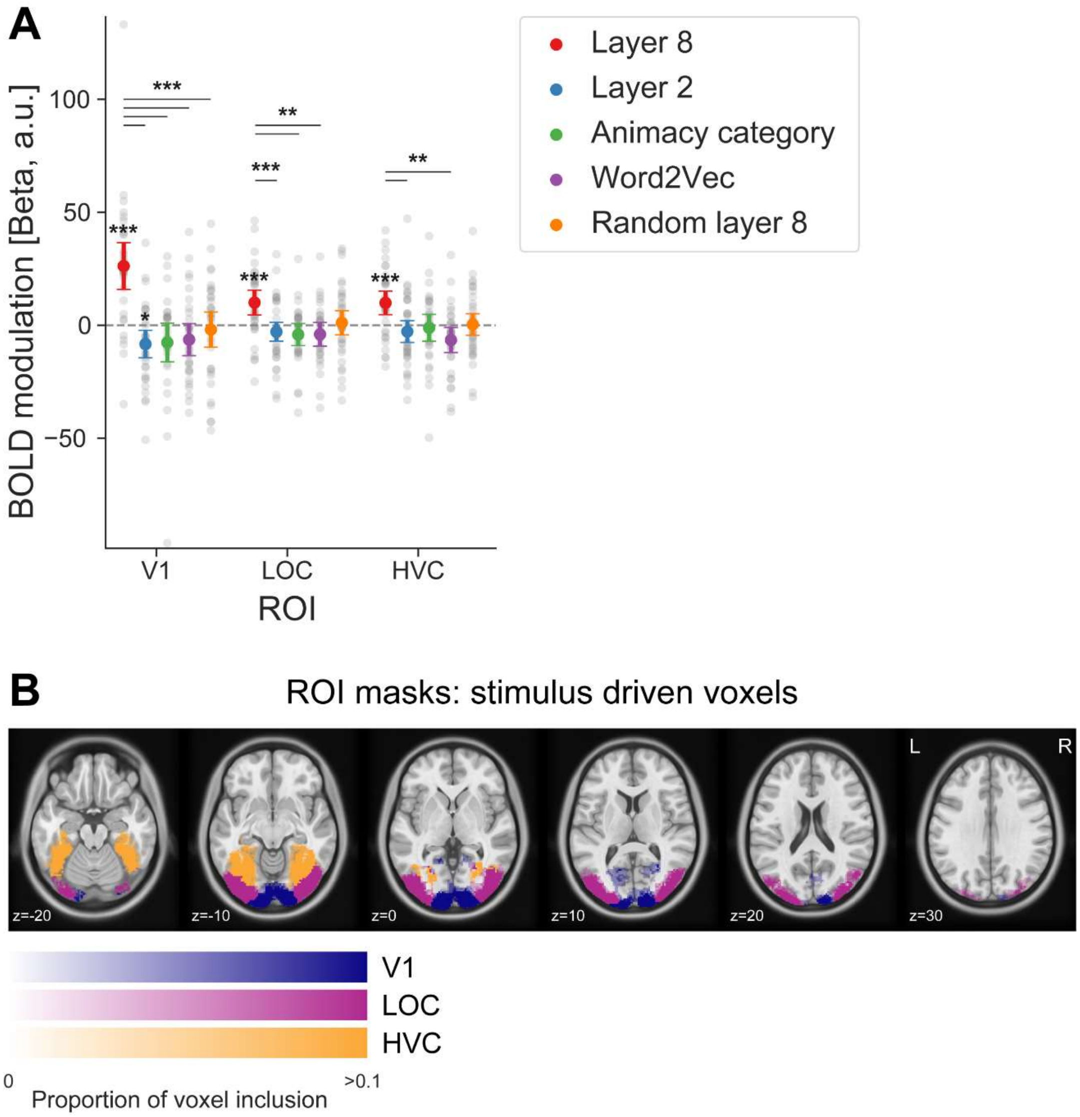

Supplementary Figure 4. Visual responses scale with high-level surprise across the visual hierarchy in stimulus-driven voxels. A) Control ROI analysis using all stimulus driven within the anatomically defined V1, LOC and HVC masks. For ROI mask creation, stimulus driven was defined as a significant activation ($z > 3.1$) of voxels by the presentation of the object images during the independent localizer run. Thus, compared to the ROI masks used in the main analyses, these ROI masks are larger, encompassing all visually driven voxels within the ROIs. Average mask size in voxels: V1 = 1254, LOC = 2443, HVC = 1383. Results show that prediction error magnitudes are best explained by high-level visual feature dissimilarity across all three

ROIs. Error bars indicate the 95% within-subject confidence intervals. Gray dots denote individual subjects. P values are FDR corrected. *** $p < 0.001$, ** $p < 0.01$. **B)** Voxels included in the ROI masks using all stimulus-driven voxels within the anatomically defined masks. Color indicates the ROI: Blue = V1, Purple = LOC, Orange = HVC. Notably, the resulting masks are significantly larger and extend further anterior in HVC compared to the original decoding-based masks. Opacity indicates the proportion of participants whose individual masks included the voxel. For visualization, full opacity corresponds to a proportion of 0.1, with voxel inclusion proportions reaching up to 1.

Finally, to enable a more detailed inspection of how our ROI masks may map onto the RSA results, we depict a whole-brain figure for each DNN layer with the three ROI masks drawn on top of the RSA results. We hope that the provided illustrations aid in better interpreting the precise location of our ROI masks. We trust that the broader activation-based ROI masks (Supplementary Figure 4; p.47), the extension of the size of the ROI masks (Supplementary Figure 5; p. 49), as well as the of the whole-brain analyses (Figure 5A,C; Figure 6A) alleviate concerns that our results are contingent on the exact ROI definitions used here.

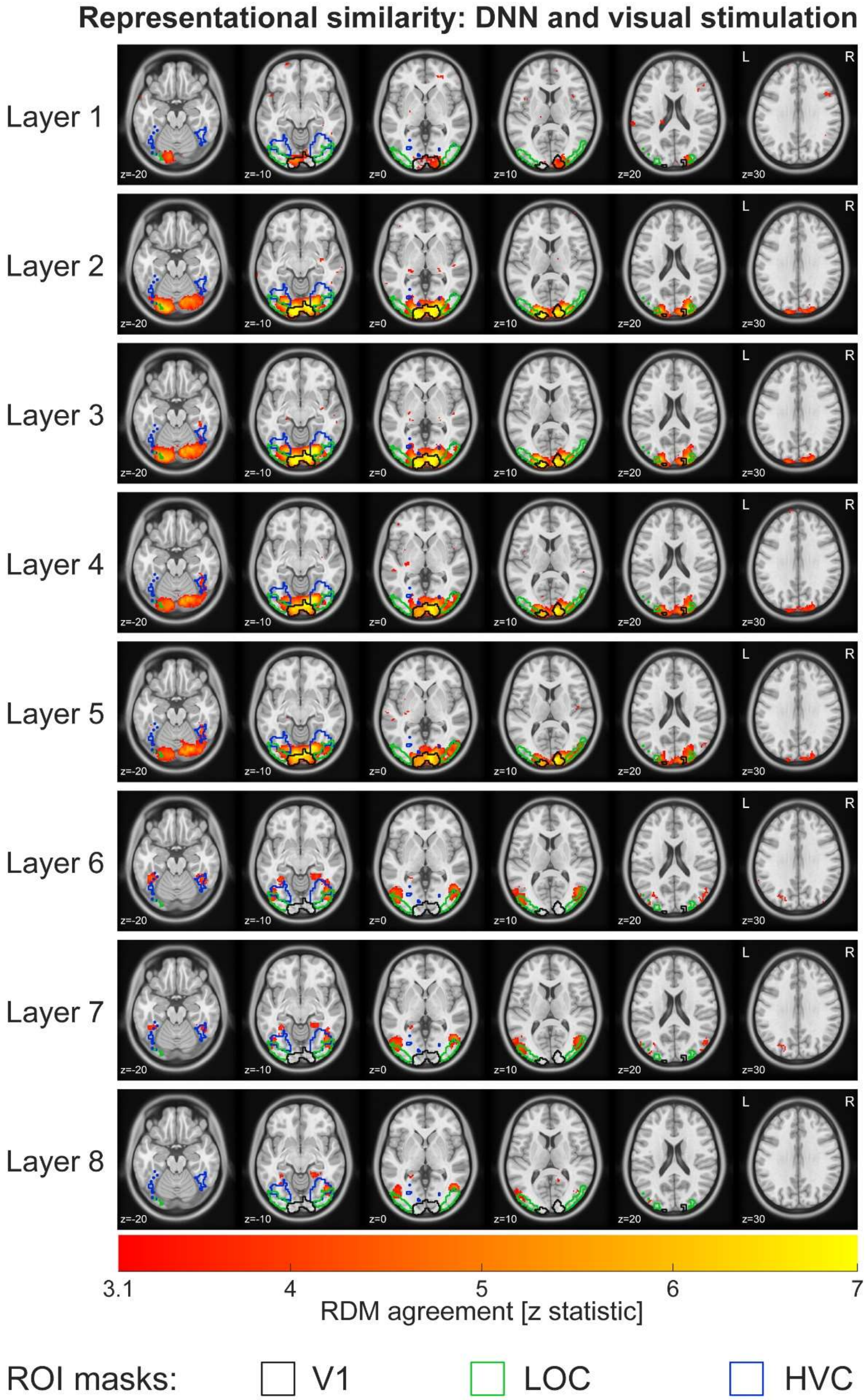

Figure depicting representational similarity analyses of visual responses showing that visual responses during the localizer were explained by a gradient of low-level to high-level visual features going up the ventral visual hierarchy. Early visual cortex (EVC) responses aligned more closely with early and intermediate DNN layers, indicative of low-level visual feature processing. Higher visual cortex (HVC) areas, like the fusiform gyrus, show a greater correlation with late DNN layers,

representing high-level visual feature processing. Red to yellow cluster indicate statistically significant correlations between the layer RDMs (layer 1-8 separately for each panel), thresholded at $z > 3.1$ (i.e., $p < 0.001$, uncorrected) of the RSA. ROI masks are illustrated as colored outlines with black = V1, green = LOC, and blue = HVC. A strong contrast between earlier convolutional layers (1-5) and later dense layers (6-8) is evident. Particularly early layers strongly map onto EVC, while later layers map onto late ventral visual areas. We also note that while our ROI definition does separate the ventral visual stream roughly into three separate stages early, intermediate and high-level visual cortex, our HVC definition does not include major anterior HVC clusters particularly correlating with late DNN layer representations. Critically, our results do not depend on the specific ROI mask definition, as larger ROI masks as those depicted here (Supplementary Figure 5) and an alternative ROI mask definition using all stimulus-driven voxels (Supplementary Figure 4) result in highly similar results as those obtained using the above depicted masks.

Third, the reviewer voices the concern that our results may be contingent on the specific layers of the DNN and therefore suggests a variation of our key analyses with all DNN layers included. In our opinion this is a critical question. To be clear, we do not believe that our results hinge directly on the specific layers 2 and 8; rather, these layers are one specific way to carve out low- vs high-level visual representations. Thus, we are sympathetic to the reviewer's suggestion to explore additional layers. However, there were multiple reasons why we chose to focus on only 2 layers. These considerations can be split into significant methodological challenges for including more layers, only limited additional theoretical information gained, and significantly complicating the readability of the manuscript. We will illustrate and support these arguments in the following paragraphs.

First, a significant problem, as also noted by another reviewer (see: R1, comment 1), is that layers of visual DNNs tend to highly correlate, thus making variance partitioning difficult if multiple, particularly similar (e.g. neighboring) layers are included in the GLM. However, we do agree with the reviewer that exploring additional layers is interesting, hence we provided the whole-brain scaling analysis results depicted in Fig5C. However, here regressions per layer were fit in separate models to the parameter estimates, instead of including all regressors in one GLM as in our primary analyses (Fig 5A,B and Fig6). This more liberal test, without considerations of variance partitioning, will necessarily overestimate the contribution of individual layers. For this reason we thresholded the figure to the most influential layer per voxel, and draw only limited conclusions from these results, stressing that Fig5C serves to "explore the landscape of prediction error modulations across DNN layers", instead focusing our interpretation on the primary analyses (Fig 5A,B and Fig6), constrained to L2 and L8.

To provide empirical support for the concern noted above, we performed the ROI analysis as suggested by the reviewer, including all DNN layers in the GLM.

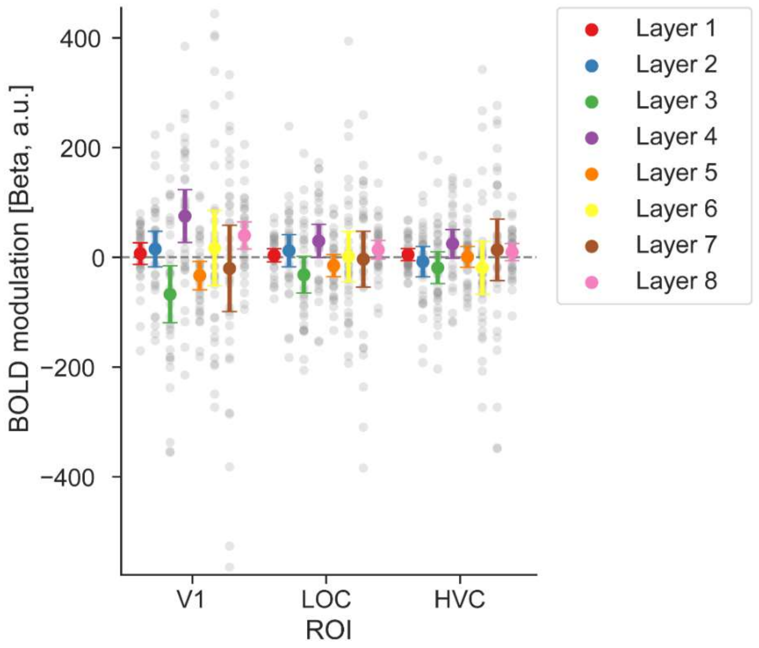

Figure depicting the ROI analysis results assessing the modulation of visual responses by surprise as indexed by feature dissimilarity across all 8 layers of the DNN. ROIs in early visual (V1), intermediate (LOC) and higher visual cortex (HVC), show no reliable results. Error bars indicate the 95% within-subject confidence intervals. Gray dots denote individual subjects.

As can be seen in the figure above, results were unreliable, with large error bars, and overlapping with zero for almost all layers. This is likely a consequence of the high collinearity between DNN layer representations. To assess whether multicollinearity was indeed a problem for the GLM including all layers as regressor, we calculated variance inflation factors, as for our main analysis following the suggestion of R1 (for details and VIF results see our reply to R1, question 1). In brief, VIFs of the regressors in the analysis including all layers substantially exceed the commonly handled threshold of >5 for all layer regressors, suggesting that variance partitioning was a substantial problem in this version of the analysis (see table below for VIF details). This is in stark contrast to the VIFs of our main analysis, including only L2 and L8 from the DNN, which yielded VIFs <2 for both layers of interest (for more detail see: Supplementary Table 2; p. 54).

**Regressor**	**Variance inflation factor**
Expected	2.2
Unexpected	2.2
No Go	1.6
Layer 1	4.1
Layer 2	**21.5**
Layer 3	**38.0**
Layer 4	**29.2**
Layer 5	**12.3**
Layer 6	**60.1**
Layer 7	**77.4**
Layer 8	**8.5**
Response category (animacy)	2.3
Word category surprise (word2vec)	3.4
Untrained layer 8 (random DNN layer 8)	1.9

Table of variance inflation factors (VIF) for each regressor of the GLM using all DNN layers. VIFs were computed on the first-level (i.e., run-level) fMRI design matrices, as supplied to FSL FEAT, and subsequently averaged across runs and participants. Obtained VIFs are very high for (almost) all regressors based on DNN layers. Note, commonly VIFs >5 (marked in bold) are considered problematic. Results suggest severe problems with variance partitioning due to collinearity of the predictors in the GLM when including all layers as regressors.

Unfortunately, given the above reasons and the VIF results, we do not believe that the model including all layer RDMs constitutes an informative analysis. Hence, we believe that this analysis should not be added to the manuscript.

Before proceeding, it is important to consider what information additional DNN layers could add to the conclusion of the study. In our view, the interpretation of our results would not change if similar results would be obtained with layer 7 instead of layer 8, or layer 3 instead of layer 2. That is, the specific layer number (approximately +/-1) does not impact our conclusions, thereby arguably limiting the theoretical usefulness of including additional layers in the analyses. As noted in the manuscript, our specific choice of layer 2 was motived by prior work, showing the largest correlation between EVC and L2 of this DNN (Mehrer et al., 2021), and considerations of fitting the GLM (i.e. using the most distant high-level layer), thereby optimizing the contrast between low- and high-level predictions.

However, we would not like the reader to misinterpret the present results as cherry-picked or specific to the exact layers of the DNN used here – rather it is important that the results are understood as concerning high-level and low-level surprise. Any other early and late layer of a visual DNN that adequately correlates with visual representations could be used and should not significantly alter the conclusions. To provide support for this claim, while avoiding the issues of variance partitioning noted above, we repeated our primary GLM based analyses (as reported in Fig. 5 and 6), using neighboring layers of those used in our primary analyses – i.e., using layers 1 and 7 in one GLM, as well as layers 3 and 7 in another GLM. Note that layer 9 was not explored, as it constitutes the class output layer of the DNN after softmax, and thus is categorical in nature.
Results showed that while minor numerical differences exist, the primary conclusions remain similar irrespective of whether layer 1, 2 or 3 was used as a low-level feature model and whether layer 7 or 8 was used as a high-level feature model. In brief, in V1 results matched those of the primary analyses, that is a reliable modulation of BOLD responses by high-level (layer 7) surprise. While qualitatively similar to the modulation of layer 8, layer 7 did quantitatively not reach the same level of statistical robustness in later visual areas, particularly in HVC. Matching the results reported in Fig. 6, both low-level surprise models tested here, layer 1 and layer 3, failed to explain prediction error magnitudes across the visual hierarchy, thus replicating the null results for layer 2. Combined these control analyses suggest that while numerical differences in the strength of the modulation exist between comparable layers (i.e. visual feature models), the conclusions remain largely unchanged by the exact choices of low-level and high-level visual surprise model. We now report these results as Supplementary Figure 7 (p. 52).

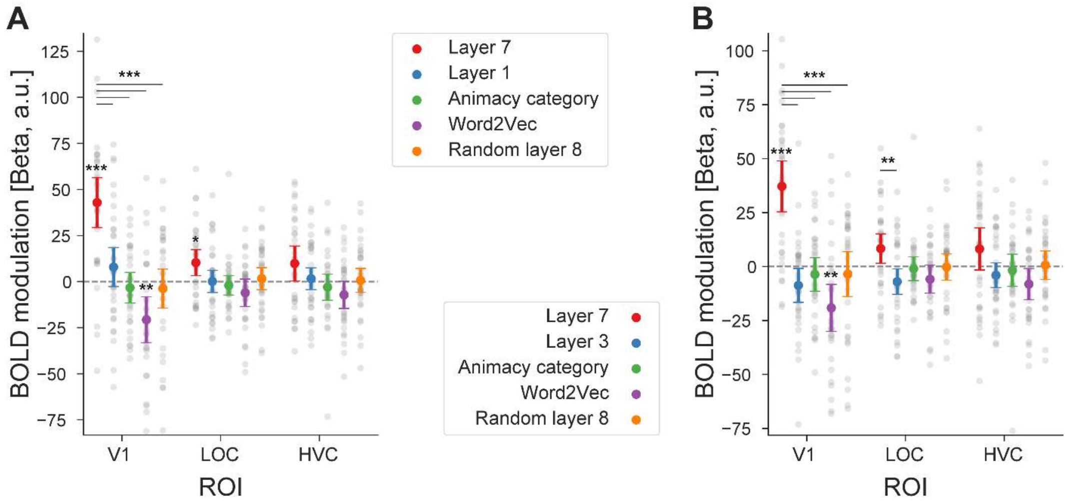

Supplementary Figure 7. Control analyses using neighboring layers of the primary prediction error analysis. To ensure that our results were not specific to the DNN layers used in the primary analyses (see the Results section for details on the layer selection), we repeated the ROI analyses using surprise as indexed by the direct neighbors of our layers of interest. **A)** ROI analysis in V1, LOC, and HVC using DNN layer 1 (blue) and layer 7 (red) as low-level and high-level surprise respectively. **B)** ROI analysis using layer 3 (blue) and layer 7 (red) surprise. Layer 9 was not explored, as it constitutes the class output layer of the DNN after softmax, and thus is categorical in nature. While compared to the primary analyses (Figure 6B), using layer 2 and layer 8 surprise, quantitative differences are evident, qualitatively the results remain identical. That is, high-level surprise (here layer 7), best explains prediction error magnitudes compared to other surprise metric, particularly in V1. In contrast to layer 8 surprise, modulations by layer 7 were not quite as pronounced in later visual areas. Like layer 2, both layer 1 and 3 surprise failed to account for modulations of visual prediction errors. Error bars indicate the 95% within-subject confidence intervals. Gray dots denote individual subjects. P values are FDR corrected. *** $p < 0.001$, ** $p < 0.01$, * $p < 0.05$.

We briefly note these results in the Discussion section as well:

> “That said, our conclusions are not dependent on layer 2 and 8 specifically; see Supplementary Figure 7 for comparable results using layer 1 or 3 instead of layer 2 as low-level, and layer 7 instead of layer 8 as high-level visual surprise model. In other words, we do not propose that layer 8 of the utilized DNN is particularly important, rather it should be seen as one instance of a high-level feature space correlating with visual representations and hence scaling with visual cortical surprise.” (line 517).

Major issue 2

The main conclusion of the paper is reflected by the following sentence 544- "We find that visual prediction errors, a key feature of hierarchical PP, primarily reflect high-level visual surprise, including in early visual cortex." This conclusion is driven by the observation that the magnitude of expectation suppression effects is best predicted by a mismatch in 'high-level visual features' encoded by layer 8, instead of the 'low-level visual features' of layer 2. However, the authors do not provide compelling support for their assumption that layer 2 measures low-level visual features while layer 8 encodes only high-level visual features. The authors show that surprise related to a mismatch in animacy nor a word-level (semantic) surprise can explain the observed relationship between 'layer 8 RDM surprise' and expectation suppression effects in the visual ROIs. However, they do not clarify what are the high-level layer 8 features that DO drive this effect.

To address this issue, the authors could, e.g., visualize the features (DNN filters, back projected to image space) of layer 8 (and for relevant additional layers, see major issue 1) that best explain expectation suppression effects in EVC. Here it is critical that the authors show that these features are more complex than low-level Gabor-like features that the EVC is thought to encode.

We agree with the reviewer that a better understanding of the features represented by the DNN is important for interpreting our results. Following the reviewer's suggestion, we visualized the features represented by the DNN layers. We now include the feature visualization results as Supplementary Fig. 1A (p. 43), including a brief description of the utilized methods in the associated figure text.
In addition to the feature visualization, we further explored feature representations in the activation maps of layer 2 and 8. Specifically, if our qualitative interpretation of the feature visualization is correct (see figure text of Supplementary Fig. 1A below), we would expect that layer 2 contains significantly more low-level visual feature information compared to layer 8, and vice versa for high-level information. To this end we performed a classification analysis, decoding object category and Gabor orientation from the activation maps. A brief methods description and results are now shown as Supplementary Fig. 1B.

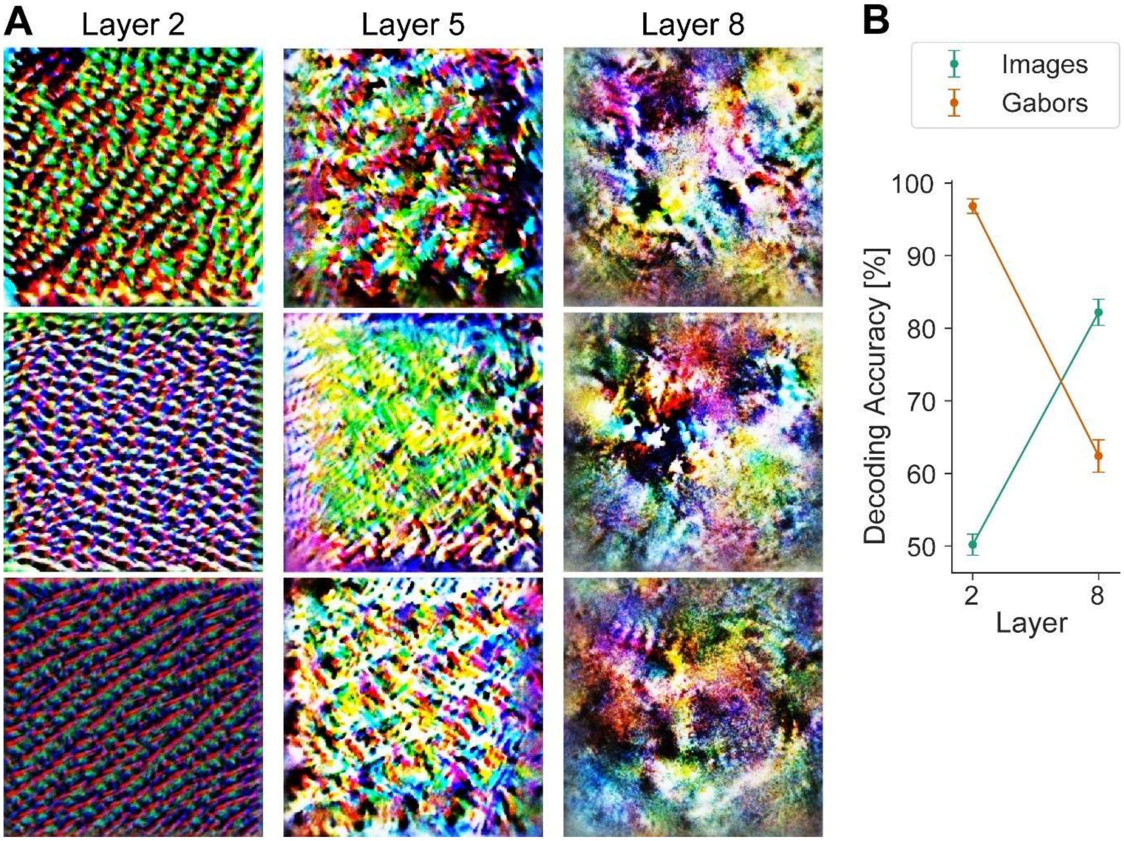

Supplementary Figure 1. DNN feature representations. A) Feature visualization using maximal activation. In brief, starting from random noise, an image was optimized to maximally activate a particular channel of a layer. The optimization function was the negative of the spatial activation of that layer and channel (for the code and more details see: https://github.com/KietzmannLab/lucid-kietzmannlab). From the resulting images, maximally driving the channel responses of the layer of interest, we can thus qualitatively interpret the kinds of features represented in that layer. Depicted are example images maximally driving activation of three channels (y axis) of layers 2, 5 and 8 (x axis). Images on the left are characterized by repeating clear orientations, thus suggesting that layer 2

represents low-level, Gabor-like features. Layer 5, in the center column, contains larger scale orientation features, but also textures. On the right side, image exemplars maximally driving layer 8, reflect more high-level visual features, such as abstract, irregular patterns and textures. Thus, the feature visualization results suggest two primary conclusions. One, layer 2 does reflect typical low-level visual features, such as orientation. Second, layer 8 reflects higher level visual features compared to previous layers, most notably complex patterns and irregular textures, with little low-level visual feature representation (e.g., no evidence of orientation tuning). Therefore, the feature visualization results support our assumption that layer 2 largely reflects low-level and layer 8 high-level visual features. **B)** Decoding of object image category and Gabor orientation from DNN layer activations. If the qualitative interpretation above is correct, we would expect that layer 2 contains significantly more low-level visual feature information compared to layer 8. In contrast, we would expect that more category information is available in layer 8 representations. We tested these hypotheses by performing two decoding analyses. First, we extracted the DNN activations of layer 2 and 8 in response to the 213 object stimuli used in the experiment. Next, we trained and tested a multi-class linear SVM, using 8-fold cross validation, to classify the 17 categories of these stimuli, thereby assessing how much category information is available to be read out by a linear classifier in each layer's activations. Second, we performed the same classification analysis using layer 2 and 8 activation maps in response to Gabor patches. Here classes were defined as Gabor orientations, spanning 0 to 169 degrees orientation, split evenly across 17 classes (orientations in steps of ~10.5 degrees). Exemplars within each class had different spatial frequencies (6 to 18 cycles), approximately matching the number of exemplars within each class in the object image dataset. Decoding accuracy, using 8-fold cross validation, is depicted on the ordinate for layer 2 and 8 (abscissa). Object category decoding (green), was significantly better in layer 8 compared to layer 2. In contrast, Gabor orientation (orange) could be decoded better in layer 2 than 8. Moreover, there was a strong interaction, with Gabor orientation being decoded substantially better than image category in layer 2, and vice versa in layer 8. In sum, these results further support that layer 2, in contrast to layer 8, preferentially represents low-level visual feature information, while layer 8 is more tuned towards high-level information, such as object categories.

In addition to the feature visualization and decoding analyses we report above, we also emphasize that the main GLM results (Fig. 6) modelled BOLD responses as a function of layer 2 and layer 8, as well as semantic (word2vec) and animacy (response) category. Word2vec and animacy category surprise likely reflect non-visual high-level surprise, but layer 8 nonetheless explained unique variance of the BOLD response, thereby suggesting that layer 8 likely reflects surprise more in terms of high-level visual features in addition to any non-visual category information captured by word2vec and animacy category. Thus, in sum, and in combination with previous work exploring representations of visual DNNs (Guclu & van Gerven, 2015; Mehrer et al., 2021; Yamins et al., 2014), we believe that our key assumptions are validated: layer 2 represents low-level and layer 8 high-level visual features.

Based on the above, we also included a summary in the Results section, when initially introducing the utilized DNN layers:

> "In addition, we explored representations of layer 2 and 8 using feature visualization and decoding techniques to further support our assumption that these layer representations serve as low-level and high-level visual feature spaces respectively. For details see Supplementary Figure 1. In brief, results showed that layer 2 represents low-level visual information, such as Gabor-like orientation features, while layer 8 has more complex texture-like (31) and categorical tuning." (line 153)

Finally, we do acknowledge that the interpretation of which precise high-level visual features are represented in late layers of a visual DNN, and indeed in high-level visual cortex, is a complicated and partially unsolved question. Arguably, neither cortical nor DNN high-level representations must conform to our preconceived linguistic categories, but future work may nonetheless succeed in charting how specific high-level feature representations may interact with the prediction error modulations seen here. However, this objective is beyond the scope of the present study.

We also added an additional paragraph to the Discussion section highlighting limitations of feature visualization and suggest relevant future work:

> "The precise features represented by visual DNNs are poorly understood. Visualizing features (Supplementary Figure 1) can help to gain an intuition for the feature tuning, but ultimately hand-crafted and better controlled feature models might be necessary to further elucidate the precise features that are reflected in visual prediction errors." (line 514)

Major issue 3

Multiple times, the authors suggest that the main effects reported could be related to more efficient processing of expected visual information in EVC (e.g., lines 398-400). Given these statements, I am surprised that the authors did not assess whether stimulus identity information in EVC, LOC and HVC response patterns increases for expected vs unexpected images, which would be consistent with de Lange's influential "less is more" finding. Such an analysis has to be performed and reported in this study, because it would allow readers to better understand how prediction error coding relates to 'efficient coding'. Also a null result, or an opposite-to-the-expected result, should be reported.

The reviewer raises an interesting point. In brief, we understand the reviewer to reason that more efficient processing of expected visual information in EVC should also result in improved (i.e., sharper) visual representations, and hence better decoding of stimulus identity for expected stimuli. While some prior work has indeed shown such a neural pattern (e.g. Kok et al., 2012), this pattern of results is by no means universally observed and different mechanisms may apply in different contexts (de Lange et al., TICS 2018). For example, Richter et al. (2018; 2022) found, using a highly similar paradigm as employed here, that expectations dampen, instead of sharpen, sensory representations. Further results in line with this dampening of object representations following statistical learning were also found in neurophysiological studies in macaque monkeys (Meyer & Olson, 2011; Kumar et al., 2017). Thus, based on prior studies it is not necessarily the case that we would expect superior decoding for expected inputs.

Second, and perhaps more importantly, more efficient processing of expected visual information does not necessarily lead to sharper representations of expected inputs as measured with fMRI. Namely, a more efficient (i.e., less resource intensive computation) could result in weaker, similar, or stronger representations than a less efficient computation, especially when examining the integral activity of a large population of neurons over hundreds of milliseconds using fMRI (Alink et al., 2018; Richter et al., 2022). In view of this interpretational ambiguity, we have now toned down our statements with respect to efficient processing, and provide a more complete explanation of possible mechanisms that could have led to our observed activity and stimulus information differences as a function of high-level surprise. As these adjustments involved changes in multiple parts of the Discussion section, we recommend reviewing the changes in the context of the manuscript. However, for completeness we also provide a list of changes here:

> "[...] one possible explanation for the observed results is that predictions allow the visual system to settle on a valid perceptual interpretation more quickly, as predictions match the bottom-up inputs. In contrast, unexpected input may require stronger (15) and more prolonged processing within the visual system to arrive at the best interpretation of the current inputs, potentially

leading to the larger BOLD signal and superior decoding of stimulus representations observed here. Put differently, our results may suggest that the larger the difference between what is expected and what is observed, at a high-level, the more cycles of recurrent processing along the ventral visual stream may be necessary to solve object recognition.” (line 421)

“Moreover, merging prediction and attention accounts, we can derive another interpretation. Following prediction error computation in higher (visual) areas, a surprise signal could be relayed down the visual system. This surprise signal, instead of constituting a prediction, may be a scalar value reflecting the magnitude of the high-level surprise (or on a predictive coding account, the precision of the high-level prediction error) and result in a gain modulation of sensory neurons in earlier areas. This surprise signal could serve to allocate attention, facilitate learning, as well as update synaptic weights and thus promote encoding of statistical contingencies following highly surprising events. Sources of this modulatory feedback could be higher visual areas that compute high-level prediction errors or the perirhinal cortex, thereby closely linking high-level surprise with novelty detection and facilitating learning (54).” (line 556)

“These results show a striking dissociation between the bottom-up tuning profile in early visual cortex and its modulation by high-level surprise. They suggest that visual surprise is computed at higher levels of the visual ventral stream and surprise signals are subsequently broadcast to earlier areas in the cortical hierarchy.” (line 569)

We also performed the decoding analysis as suggested by the reviewer. In brief, for each participant and within each ROI we extracted the single-trial parameter estimates (BOLD) from the localizer runs. On this data we trained an 8-class classifier, with the 8 classes corresponding to the 8 objects seen by the participant. Next, we tested the classifier on the single-trial parameter estimates from the main task runs. To improve SNR, we averaged the single-trial estimates for each object and expectation condition (expected or unexpected) within runs. Finally, we averaged the obtained decoding accuracies across runs to obtain a mean decoding accuracy for expected and unexpected trials per participant. On average decoding of the object stimuli was well above chance (12.5%), but did not reliably differ between expected and unexpected stimuli in any of the three ROIs: HVC (Expected: 47.5%, ±3.0%; Unexpected: 46.9%, ±3.0% [mean, ±95%CI]), LOC (Expected: 63.1%, ±2.2%; Unexpected: 64.5%, ±2.6%), V1 (Expected: 73.1%, ±3.3%; Unexpected: 73.8%, ±3.3%).

**ROI**	**Test statistic**	**P value**
V1	$t_{(32)} = -0.51$	$p = 0.613$
LOC	$t_{(32)} = -1.02$	$p = 0.315$
HVC	$t_{(32)} = 0.55$	$p = 0.588$

Finally, it is important to stress that contrasting the visual representations of expected compared to unexpected trials was not the focus or objective of the present study. Instead, our key analyses investigated how surprise modulates sensory responses elicited by different unexpected stimuli that differed in terms of their amounts of surprise (i.e., the analyses only consider the unexpected trials). Thus, inspired by the reviewer’s suggestion to perform a decoding analysis, we tested how visual representations (decoding performance) might be modulated by high-level surprise. In brief, we performed an analysis similar to the regression of BOLD responses on high-level surprise (Fig. 5D) but using a multivariate outcome metric (decoding performance) instead. We first trained multi-class SVMs (8 object stimuli as classes) for each participant using the prediction-free localizer data. Next, we predicted class membership on the trial level during main task. Finally, per participant, we regressed the true class probabilities derived from the decoder onto high-level (layer 8) surprise. In line with our univariate results, multivariate results showed that decoding performance significantly scaled with high-level surprise in V1 ($p<0.001$) and HVC ($p<0.001$), albeit not reliably in LOC ($p=0.355$). Therefore, within V1 and HVC the acuity of visual representations seemed to increase the more surprising a stimulus was in terms of high-level (layer 8) features. These results are in line with a dampening of neural responses inversely scaling with surprise and thereby support previous studies reporting a dampening

by expectations (Meyer & Olson, 2011; Kumar et al., 2017; Richter et al., 2018; 2022). We added these results as Fig. 5E (p. 11).

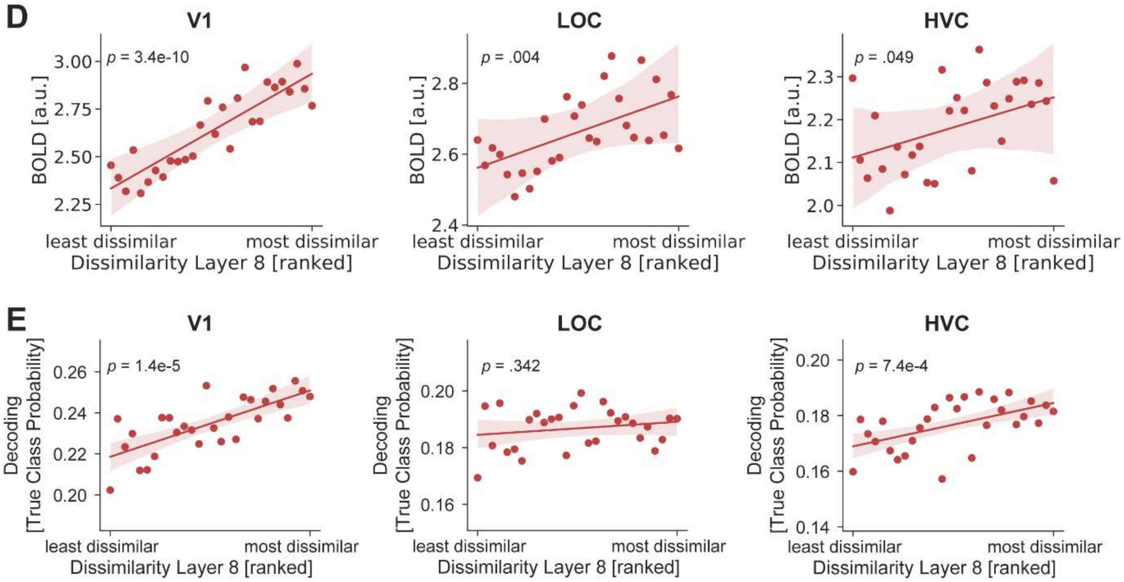

Figure 1. […] D,E) ROI analysis regressing BOLD responses (**D**) or decoded true class probability (**E**) onto high-level visual dissimilarity. Results indicate a monotonic relationship between high-level surprise and BOLD responses across all three ROIs, as well as decoding performance in V1 and HVC. The chance level for decoding the true class probability is 0.125. For display purposes dissimilarities were ranked and averaged across participants, while regression models were fit per participant on the correlation distances. (line 270)

We also discuss these results in the Results section:

"In addition to modulating BOLD responses, it is possible that high-level surprise may also result in sharper visual representations. To test this, we performed a decoding analysis, trained on the independent localizer data and tested on the main task data. Decoding accuracy of the object images (for details see: *Materials and Methods: Statistical Analysis: Decoding as function of surprise*) increased with layer 8 dissimilarity in V1 ($t_{(32)} = 5.50$, $p < 0.001$, $d_z = 0.96$, mean $r = 0.08$) and HVC ($t_{(32)} = 3.89$, $p < 0.001$, $d_z = 0.68$, mean $r = 0.05$), but not LOC ($t_{(32)} = 0.96$, $p = 0.342$, $d_z = 0.17$, mean $r = 0.07$). In other words, the more surprising an unexpected stimulus was in terms of high-level features, the better it could be decoded from V1 and HVC. To summarize, visual responses and the fidelity of the visual representations appeared to monotonically increase with high-level visual surprise across major parts of the ventral visual system." (line 295)

Relevant section from the Discussion:

"Additionally, the more surprising objects were in terms of high-level features, the better they could be decoded by a linear classifier. Therefore, our data suggested that both the magnitude but also the stimulus information contained in the visual response scaled with high-level surprise. This may imply a possible functional role in enhancing visual representations of inputs that do not match our high-level predictions of the world." (line 395)

And we describe the analysis in more detail in the Material and Methods:

"**Decoding as function of high-level surprise**

In addition to the univariate analysis, we regressed decoding performance onto high-level (layer 8) surprise. Specifically, for each ROI and participant we trained a multi-class SVM on the single trial parameter estimates from the prediction-free localizer data. Next, we predicted class

membership probabilities on a per-trial basis during the main task using single trial parameter estimates of all unexpected image trials. Finally, per participant, we regressed the true class probabilities derived from the decoder onto high-level (layer 8) surprise. Across participants we performed inference by submitting the obtained beta coefficients to a one-sample t-test. A significant positive association would thus suggest that decoding performance increases with layer 8 dissimilarity." (line 928)

Major issue 4

In the discussion, the authors acknowledge that attention and arousal effects cannot not be conclusively ruled out as an explanation for expectation effects. The authors, however, do not clearly highlight an aspect of the experimental paradigm that is of particular importance to this discussion.

Specifically, the task during expected trials 1) requires less visual attention and 2) involves a much simpler decision and motor preparation process as compared to unexpected trials. Hence, during expected trials participants can prepare a motor response as soon as they hear the sound cue, and then they simply execute it, as nothing unexpected happens. In contrast, during unexpected trials, participants will need to pause their prepared motor response, pay close attention to the image and then, possibly (~50/~50), abort their prepared response and initiate and execute a new motor response. Such differences between the main conditions appears to be consistent with the reported enhanced responses to unexpected image in the precentral gyrus.

I agree that an attention/arousal/decision making effect does not provide a good explanation for the layer 8 surprise modulation for expectation suppression in EVC.

We do consider the question of attention and arousal relevant for the debate, as is perhaps also evident from our discussing the reviewer's perspective paper on the matter. We agree with the reviewer that there are clear differences between expected and unexpected trials, which have also been discussed in detail elsewhere (Alink & Blank, 2021; Richter & de Lange, 2019; Richter, 2021). Critically, these differences do not necessarily pertain to the present analyses and conclusions. We like to emphasize that our primary analyses (Fig. 5-6) do not contrast expected and unexpected stimuli, but only rely on stimulus differences between the various unexpected stimuli. Thus, all trials contributing to the key analyses, and hence to the conclusions presented here, were unexpected image trials, thereby avoiding the major pitfall noted by the reviewer related to the contrast of expected vs unexpected trials. In fact, we believe that this aspect is a major strength of our analysis approach, allowing inferences beyond previous work. We now better clarify this in our manuscript (line 538):

> "First, attention accounts (49) emphasize that generic prediction error-like responses may arise due to the more demanding processing associated with responding to unexpected compared to expected inputs. While constituting a viable account of previous studies, it is critical to emphasize that the results reported here exclusively analyzed differential responses to unexpected inputs, and thus are not affected by differences in processing demands associated with responding to expected compared to unexpected inputs."

We nonetheless concede that there might be differences in arousal/attention between different unexpected inputs, a topic we will return to later in reply to another comment by the reviewer.

However, two other arguments made against an attention-based explanation are not compelling.

First of all, the authors find that expectation effects are stronger in earlier visual areas, and they argue that this rules out an attention-based explanation because attention effects should increase from lower to higher level brain areas. However, this argument hinges on two electrophysiological studies in animal models. I would like to stress here that the work of Logothetis (What we can do and what we cannot do with fMRI, 2008), has taught us that one cannot simply assume that spiking rate effects

translate into comparable BOLD effects: "page 876- Yet, as I have indicated above, the BOLD signal is primarily affected by changes in excitation-inhibition balance, and this balance may be controlled by neuromodulation more than by the changes in spiking rate of a small set of neurons. In fact, the BOLD signal is strongly modulated by attention…".

This is an excellent point and caution is indeed warranted when interpreting BOLD signal data, and our argument indeed primarily hinged on two electrophysiological studies. Moreover, fMRI BOLD studies, charting the modulation of neural responses in visual cortex by attention, are less clear cut. Therefore, we agree that the fMRI BOLD evidence does not clearly support our previous statement of “even more pronounced [modulations of attention] in higher areas” and accordingly have removed it from the manuscript.

Second, the argument that an expectation-based explanation is more parsimonious is inconsistent with the recent claim by Richter and de Lange that expectation effects are gated by attention (D Richter, FP de Lange - eLife, 2019). Hence, given the authors previous arguments and findings, an expectation-based explanation of expectation suppression requires an attention component, and therefore a pure and parsimonious expectation-based explanation does not exist.

The reviewer raises an important point. However, we would contextualize our prior work (Richter & de Lange, 2019) slightly differently, and would like to emphasize the difference between attention *gating* predictions and the modulations in visual cortex reflecting attention itself. In our opinion our prior work shows the former, i.e. modulations by stimulus surprise are only observed when participants pay attention to the stimuli. That said, while we think that this is a relevant debate, it may be beyond the scope of the present manuscript, particularly because the present analyses do not depend on contrasting responses to expected and unexpected stimuli. However, we concede that even between different unexpected stimuli attention and arousal could differ. Therefore, we included this consideration in the discussion.

It is important to us that our manuscript fairly reflects the reviewer’s perspective. Hence, we have improved the relevant sections of the Discussion section and included our prior study cited by the reviewer:

> “However, previous work showed that predictions are gated by attention (10), thus suggesting that both attention and prediction are necessary for the here observed modulations”. (line 550)

Indeed, we believe that our prior work cited by the reviewer does provide additional support that at least part of the generic prediction error effects are due to attention or arousal changes. Accordingly, we expand on this in the discussion section:

> “In line with this interpretation, this prior study also suggests that both stimulus specific and unspecific modulations contribute to prediction errors, with the latter likely reflecting generic changes in arousal or attention (10)” (line 551)

We have also toned down the associated conclusion, and incorporated an attention-based explanation into one of the two primary interpretations we offer in the updated manuscript.

> “On either account, it is likely that the present modulations in EVC reflect a consequence of the downstream surprise detection in later (visual) areas.
>
> Moreover, merging prediction and attention accounts, we can derive another interpretation. Following prediction error computation in higher (visual) areas, a surprise signal could be relayed down the visual system. This surprise signal, instead of constituting a prediction, may be a scalar value reflecting the magnitude of the high-level surprise (or on a predictive coding account, the precision of the high-level prediction error) and result in a gain modulation of sensory neurons in earlier areas. This surprise signal could serve to allocate attention, facilitate learning, as well as update synaptic weights and thus promote encoding of statistical contingencies following highly surprising events. Sources of this modulatory feedback could be

> higher visual areas that compute high-level prediction errors or the perirhinal cortex, thereby closely linking high-level surprise with novelty detection and facilitating learning (54)". (line 553)

We hope that we have thereby adequately responded to the point raised by the reviewer.

Other issues:

- In this paper reaction times are shown to increase with layer 8 surprise. It is unclear, however, whether or not this is related to category effects (or semantic effects). For completeness, it would be good if the authors report this.

We agree with the reviewer that response category (animacy) effects are important to assess when considering behavioral responses. Indeed, independent of the scaling with layer 8 surprise splitting behavioral responses according to animacy category is a valuable addition. It is essential to note however that animacy category and the need to adjust button presses are highly colinear. However, it is possible to delineate response adjustment effects from more perceptual decision effects by splitting the behavioral data (RT and accuracy, for completeness) for unexpected trials into two types: (1) unexpected images requiring the same response as the expected stimulus (hence not requiring a response adjustment compared to the predicted category) and (2) unexpected images requiring a different response (i.e. involving a response adjustment). Based on previous studies (Yan et al., 2023), we would expect RT decrements for both – i.e., $RT_{exp} < RT_{unexp\_same} < RT_{unexp\_different}$. This is indeed what we observed in the data, now reported as Fig. 2, with the associated updated description of the results:

> "Participants learned and used the underlying statistical regularities to predict inputs thereby facilitating performance (Figure 2), in terms of response times (RT; $F_{(1.4,43.3)} = 23.5$, $p < 0.001$, $\eta_p^2 = 0.42$) and response accuracy ($F_{(1.4,46.3)} = 7.8$, $p = 0.003$, $\eta_p^2 = 0.20$). Specifically, RTs to expected images (501ms) were faster compared to unexpected images requiring the same button press (509ms; $t_{(32)} = 2.41$, $p = 0.019$, $d_z = 0.14$) and different button press (524ms; $t_{(32)} = 6.77$, $p < 0.001$, $d_z = 0.39$). The former contrast demonstrates that surprising stimuli resulted in slower responses even when the stimulus required the same response (i.e., was of the same animacy category) as the expected input. Additionally, adjusting responses resulted in even slower RTs (unexpected same vs unexpected different: $t_{(32)} = 4.36$, $p < 0.001$, $d_z = 0.25$). Response accuracy only showed a reliable decrements when unexpected stimuli required a different response, thus likely reflecting response errors due to invalid prediction (expected vs unexpected different: $t_{(32)} = 3.75$, $p = 0.001$, $d_z = 0.61$; expected vs unexpected same: $t_{(32)} = 0.79$, $p = 0.43$, $d_z = 0.13$; unexpected same vs unexpected different: $t_{(32)} = 2.97$, $p = 0.008$, $d_z = 0.48$). Overall behavioural facilitation thus demonstrated that participants benefitted from prediction." (line 111).

Figure 2 (p. 5) was updated accordingly:

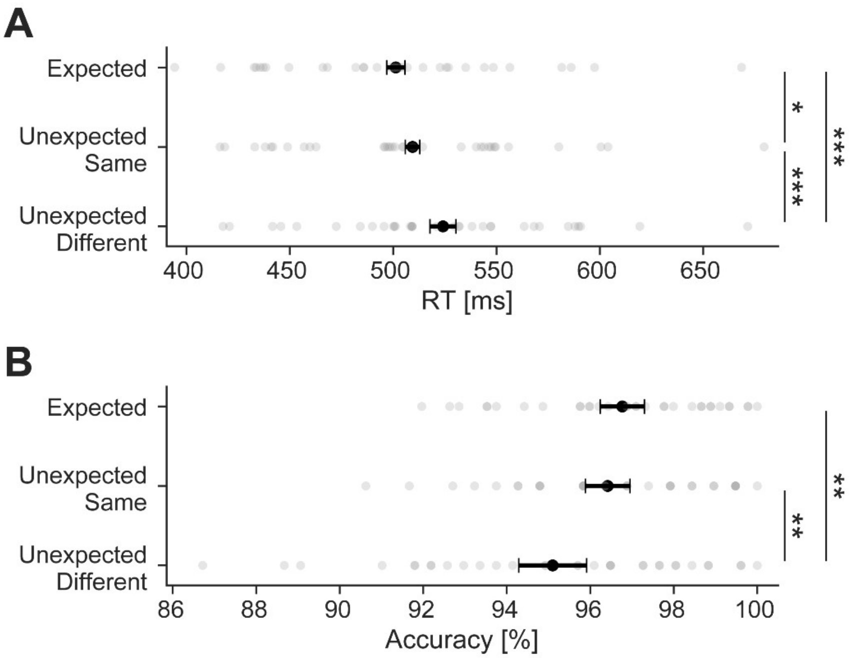

Figure 2. Behavioural facilitation due to prediction. A) Reaction times (RT) to expected objects were faster compared to unexpected images requiring the same or different response as the expected stimulus. **B)** Accuracy of responses was high overall, but responses were more accurate to expected compared to unexpected images requiring a different response than the expected stimulus. *** p < 0.001, ** p < 0.01, * p < 0.05.

The corresponding method section has also been updated (line 696):

> "Additionally, unexpected trials were split into trials requiring the same button press as the expected image (unexpected same) and trials requiring a different response (unexpected different). RT and accuracy data were analyzed using one-way repeated measures ANOVAs with three levels. Following significant results, post hoc t-tests were conducted, and p-values corrected using the Holm-Bonferroni method."

Finally, following the reviewer's suggestion, we also added animacy category and the word2vec derived (semantic) dissimilarities to the multiple regression RT analysis. Results, depicted below, now show that only animacy category surprise reliably explains RT increases, while layer 8 surprise no longer explains significant unique RT variance. Thus, in contrast to the fMRI BOLD results animacy category is the sole contributor to RT increases. Therefore, we removed the previous report of a significant modulation of RT with layer 8 surprise from the manuscript. For a summary of the associated test statistics see the table below.

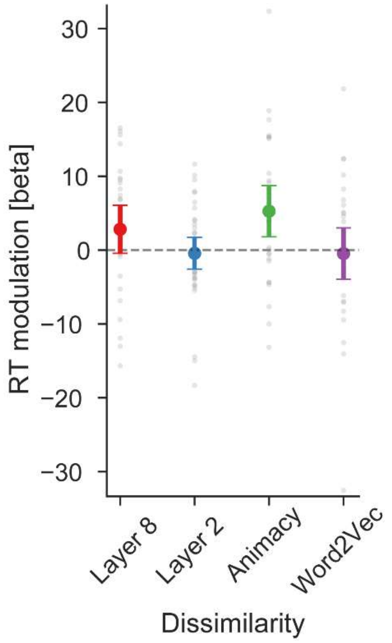

Figure. Behavioural response slowing correlates with animacy category surprise. Behavioral responses were slower when stimuli were of the unexpected animacy category. Neither high-level, low-level or word2vec surprise explained additional decrements in RT. Depicted are the slope coefficients (betas) regressing RT onto layer 8 (red), layer 2 (blue), animacy (green), and word2vec (purple) dissimilarity.

Contrast	Test statistic	P value	Effect size
Layer 8	$t_{(32)} = 1.96$	$p = 0.12$	$d_z = 0.34$
Layer 2	$t_{(32)} = -0.36$	$p = 0.97$	$d_z = -0.06$
Animacy category	$t_{(32)} = 3.24$	$p = 0.01$	$d_z = 0.56$
Word2Vec	$t_{(32)} = -0.28$	$p = 0.78$	$d_z = -0.05$

Table. Results of one sample t-tests contrasting RT modulations against zero. Only animacy category had a significant effect on RT scaling.

- in Figures 3 and 5, layers 1-8 colours should not be presented on a continuous colour scale, as it is a discrete variable (there is no layer 2.5). A single colour block per layer would make it much easier to find out which blob relates to which layer

We thank the reviewer for this helpful suggestion. We have corrected this oversight and have improved Fig. 3A (p. 7) and Fig. 5B (p. 11) accordingly.

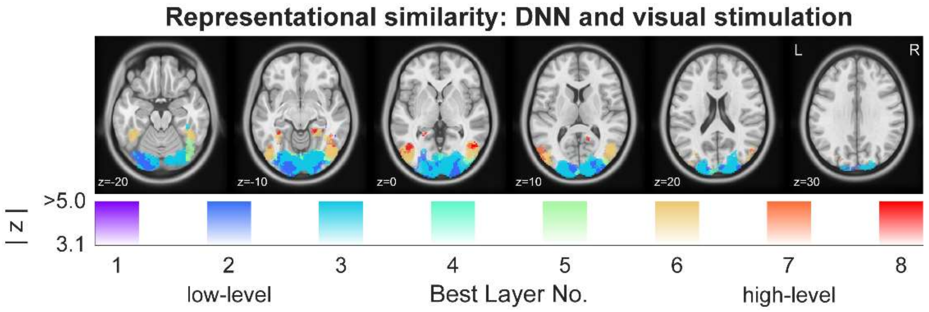

Figure 3A.

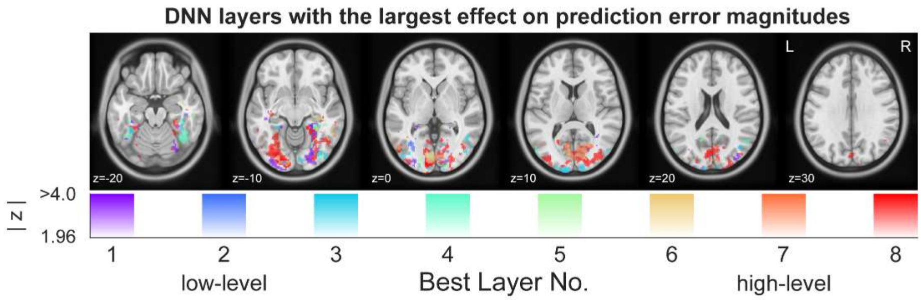

Figure 5B.

- Figure 4: the lower figure depicting the main effect (scatter plot) is very difficult to understand because: the x-axis is not labelled (z-scored corr. dist), the y-axis is not labelled (unexp-exp modulation). Moreover, the authors should explain in the legend why the dots are arranged in lines (resolution of the RDMs?)

We apologize for any technical issues, as the axes of the scatter plot in Fig. 4 were labelled in the uploaded pdf (x-axis = Dissimilarity; y-axis = BOLD [a.u.]). However, in addition to the technical problems, we believe that the description of the analysis rationale could be improved, in view of the reviewer's comment. As explained in our reply to major issue 4, it is important to emphasize that our analyses do not contrast unexpected vs expected trials (unexp-exp), but only use unexpected trials. It is a regression of the BOLD response as a function of surprise. Hence, we improved the relevant sections of figure text, the Materials and Methods, as well as Discussion sections to clarify this critical detail. Additionally, we now specify in the figure text of Fig.4 that, as speculated by the reviewer, the dots are indeed arranged in lines because of the resolution of the RDMs.

Figure 4 figure text:

> "**B)** Analysis procedure. The left side shows a single trial with a letter cue predicting a specific image. Below multiple unexpected images are illustrated, which were presented on other trials. The right side depicts the extraction of low-level and high-level visual feature dissimilarity from layer 2 (low-level) and layer 8 (high-level) of the visual DNN. Dissimilarity of the unexpected seen image relative to the expected stimulus was then added as a parametric modulator in the first level GLMs of the fMRI analysis. For illustration purposes, the graph at the bottom uses data from one example participant to depict how BOLD responses (example data from V1; ordinate) are modulated as a function of low-level (blue) and high-level (red) visual dissimilarity (abscissa). A positive slope thus indicates that the more dissimilar a seen image was relative to the expected stimulus the more vigorous the neural response. Dots indicating individual dissimilarities (i.e., distances of seen unexpected images compared to the expected image) are arranged in rows, reflecting the granularity of the available surprise distances for this example participant. In the example data, the image of the unexpected woman would hence result in larger prediction errors compared to the image of the unexpected guitar, because of the larger high-level surprise. Critically, this analysis only uses BOLD data from unexpected image trials. Additional control variables for task relevance (animacy) and word meaning, discussed in more detail later, were also included. We performed this parametric modulation analysis in a voxel-wise fashion across the whole brain." (line 183).

Relevant excerpt from the Materials and Methods: Statistical Analysis: fMRI data analysis:

> "Importantly, while the distance metric (surprise) thus depends on the distance of the seen surprising image from the expected (unseen) image on each trial, the parametric modulation only concerns trials with unexpected images. Thereby this analysis does not contrast fMRI BOLD responses to unexpected compared to expected inputs, but exclusively assesses the scaling of unexpected images by surprise." (line 794).

And from the Discussion section:

> "While constituting a viable account of previous studies, it is critical to emphasize that the results reported here exclusively analyzed differential responses to unexpected inputs, and thus are not affected by differences in processing demands associated with responding to expected compared to unexpected inputs." (line 541).

- Related to the above point, the authors perform linear regression for their main analysis of layer8-exp suppression modulation. However, based on the 4b plot, it is not possible to see whether the linearity assumption is valid. The authors should statistically assess whether their linearity assumption is justified.

Fig. 4b was only intended to illustrate the analysis rationale, using example data from one ROI of one participant. Because the analysis is performed per participant and voxel, visual inspection to confirm the assumption of linearity appears impossible. As noted in our reply to the previous point, we have improved the figure text of Fig.4, particularly for Fig4b to avoid confusion.
Moreover, we note that we test our primary hypothesis using two complementary analysis approaches. In the first approach, we use surprise (as derived from the DNN layers) as a parametric modulator in a GLM (Fig 5A/B, Fig6) – for details see: Materials and Methods: Statistical Analysis: fMRI data analysis. In the second approach, we use linear regressions (Fig 5C), as described by the reviewer above – for details see: Materials and Methods: Statistical Analysis: Regression of BOLD onto dissimilarity.
We tested the assumption of linearity for the second analysis approach, "Regression of BOLD onto dissimilarity" for the ROI analyses. First, we fit the standard linear model and a 2nd order polynomial model. We then compared the fit of the two models. If the polynomial model significantly improved the fit, it suggests a potential violation of the linearity assumption. As models are fit per participant, we here report the number of participants (of total n=33) for who the polynomial model better fit the data per ROI. V1, n=3; LOC, n=2; HVC, n=1. Thus, results suggest no widespread violation of the assumption of normality.

Reviewer #3:
1) Expectations, or predictions, influence our perception, but the underlying neural mechanisms are unclear. This study investigates how prediction errors are processed across visual cortex, using FMRI and representational distances based on a deep neural network (DNN). It is reported that high-level prediction errors are found in early visual cortex, and argued to better fit proposals in which prediction error processing reflects higher-order cortical rather than local feature representations, consistent with recent work in non-human primates (e.g., Uran et al., 2022 Neuron). The FMRI and computational methods appear solid, but I think a number of questions should be addressed, to clarify interpretations of the results, related to how low-level and high-level features have been defined, the behavior and FMRI data in the prediction-free condition. Please see specific comments below.

We thank the reviewer for the helpful comments. Indeed, many comments resemble questions and recommendations also made by the other reviewers, particularly reviewer 2. Thus, below we will frequently refer to our replies to R2 – to be clear, this is not because we believe that R3's comments are less important, but simply because we addressed the reviewer comments in chronological order.

Specific comments:

2) The definition of low-level vs high-level features were derived from DNN layers. However, it is not clear from the stimulus examples given (Fig. 4A), to what degree that low-level and high-level features defined in this way correspond to the common usage of these terms in vision research. For example, "high-level" included quite detailed (low-level) features in the examples, and "low-level" seems to carry overall gist or object identification (high-level). Perhaps this could be clarified in the main text? In response to a similar question from R2 (see our reply to R2, Major issue 2), we now include a visualization of the features layer 2 and layer 8 reflect. We hope that this visualization and the associated discussion help in the interpretation of the feature levels.

3) Further, in Fig. 4B, the building versus hand differ markedly in low-level features, seemingly more than the difference in guitars. However, if I am interpreting the color-code correctly, the representational dissimilarity matrix (RDM) for low-level features shows a considerably smaller difference for building versus hand (compared with the guitars). So what is the DNN picking up on? Please refer to our reply above to question 2 and to R2's Major issue 2 for more details on what the DNN might be picking up on. In addition, we like to emphasize that the depicted RDM in Fig. 4B is merely one example RDM of one set of 8 stimuli seen by one participant. Other participants saw different images and hence the results reported here are not contingent on this one RDM. We now stress this aspect of our design in the Discussion section:

> "We made this choice to rule out, by design, that specific images or cue-image pairs drive the results, for example due to high representational similarity for some cue-image pairs. Moreover, because participants were shown different stimuli (eight selected from a set of 213 stimuli), our results are unlikely to be contingent on any specific set of eight stimuli." (line 621)

4) The large high-level surprise example in Fig. 4A differs in both high- and low-level features compared with the expected object. To help with controlling for differences at multiple levels, were stimuli used where low-level features were matched but only high-level features differed? It would be useful to present cases of such stimuli.

We agree with the reviewer that any stimulus resulting in high-level surprise will entail some low-level surprise as well. Indeed, without low-level differences the seen image is necessarily identical to the expected one. Our analyses assessed differences in surprise *magnitude* across the different levels of description (layers), which we tried to illustrate with the examples shown in Fig. 4 (p. 8). We have improved our description of the analysis rationale (also in reply to a related comment by R2; line 183). We believe that the improved figure text better explains the rationale. Moreover, our key analyses included both layer 2 and 8 in the same GLM, only attributing unique explained variance to each regressor. Additionally, to prevent similar concerns for our readers, and as also noted in our reply to question 3 above, we now stress that our results do not depend on specific example images in Fig4A (see our reply to comment 3 above), but that those images only constitute 8 of 213 images used in the experiment.

5) RE: Fig. 5. Is it possible that layer 8 may include high-level surprise as well as low-level surprise elements (considering the example images provided in the paper)? That is, is it possible, for instance, that layer 2 includes low-level surprise and layer 8 includes low-level and high-level surprise, which might lead to a greater prediction error overall for the latter? Could this contribute to the differences seen in Fig. 5B, where V1 shows the highest modulation for layer 8 (responding to both high and low levels), whereas LOC and HVC show less modulation (responding only to high levels)?

We thank the reviewer for raising this important point. As noted above any stimulus eliciting high-level surprise must contain some low-level surprise, as without low-level differences it would be the same

image. However, our key analyses included surprise as measured by L2 and L8 in the same GLM, thus any variance not uniquely associated with L8, but also explained by L2 (low-level) surprise, will not contribute to the observed results. We now emphasize this important point when initially introducing our modelling approach:

> "To model how the BOLD response changed as a function of how visually surprising an unexpected stimulus was in terms of low-level (layer 2) and high-level (layer 8) visual features respectively, we used the dissimilarity of each surprising image, compared to the stimulus expected on that trial, as parametric modulators in the fMRI GLM analysis (for details see *Materials and Methods: Data analysis*). Using this approach, variance not uniquely associated with either regressor will not contribute to the observed results. Thus, in addition, we included multiple control variables, such as task-relevant stimulus animacy and word-level (semantic) dissimilarity." (line 214).

While we concede that this modelling approach only accounts for the low-level variance explained by layer 2, we stress that the RSA results showed that layer 2 best explained visual responses during the prediction-free localizer in major parts of posterior visual cortex (see Fig. 3). This demonstrates that layer 2 is an adequate low-level visual feature model (also see Mehrer et al., 2022). Moreover, layer 2 (low-level visual surprise) did not explain any prediction error modulation in V1, implying that low-level features did not contribute to the observed activity modulations (for control analyses using layer 1 and layer 3 as low-level surprise see Supplementary Figure 7). Combined, these results suggest then that it is unlikely that layer 8 scaled with V1 prediction errors because of a combination of high-level and low-level surprise.

6) RE: Fig. 3. As currently presented, it appears that early visual cortex may correspond to DNN layers 1-4-ish and higher visual cortex to DNN layers 5-8-ish. So it would be useful to show ROIs in addition to the current DNN layer color code, to be able to more specifically gauge how DNN layers relate to the ROIs.

This is an excellent point, which we addressed in response to a similar concern by R2 (see R2, major issue 1 for more details). For convenience, we copy the relevant figures also below here. We note that drawing the ROI masks on the multi-colored Fig 3 was impossible, given the lack of clearly distinguishable colors. Thus, we chose to provide the ROI map in an additional panel added to Fig. 3 as panel B (p. 7).

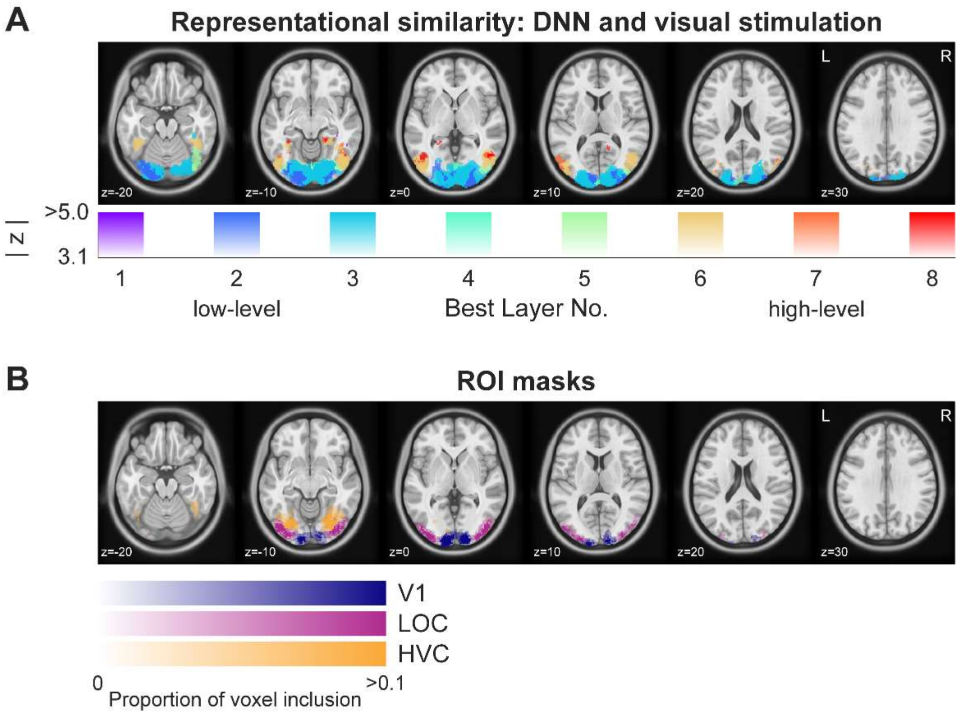

Figure 3. Convergence of DNN and visual cortical representations showing a gradient from low- to high-level features in prediction-free contexts. A) Representational similarity analysis of visual responses shows that (feedforward) visual responses during the prediction-free localizer were best explained by a gradient of low-level to high-level visual features going up the ventral visual hierarchy. Early visual cortex (EVC) responses aligned more closely with early DNN layers, indicative of low-level visual feature processing (depicted in cold colours: purple to blue). Higher visual cortex (HVC) areas, like the fusiform gyrus, show a greater correlation with late DNN layers, representing high-level visual feature processing (illustrated in warm colours: yellow to red). Analysis was masked to visual cortex and thresholded at $z > 3.1$ ($p < 0.001$, uncorrected) of the RSA. **B)** ROI masks, depicting voxels included in the anatomically and functionally defined masks. Color indicates the ROI: Blue = V1, Purple = LOC, Orange = HVC. Opacity indicates the proportion of participants whose individual masks included the voxel. For visualization, full opacity corresponds to a proportion of 0.1, with voxel inclusion proportions reaching up to ~0.7.

In addition, we now provide a per layer figure with the RSA results corresponding to Fig 3 with the ROI masks drawn over it in.

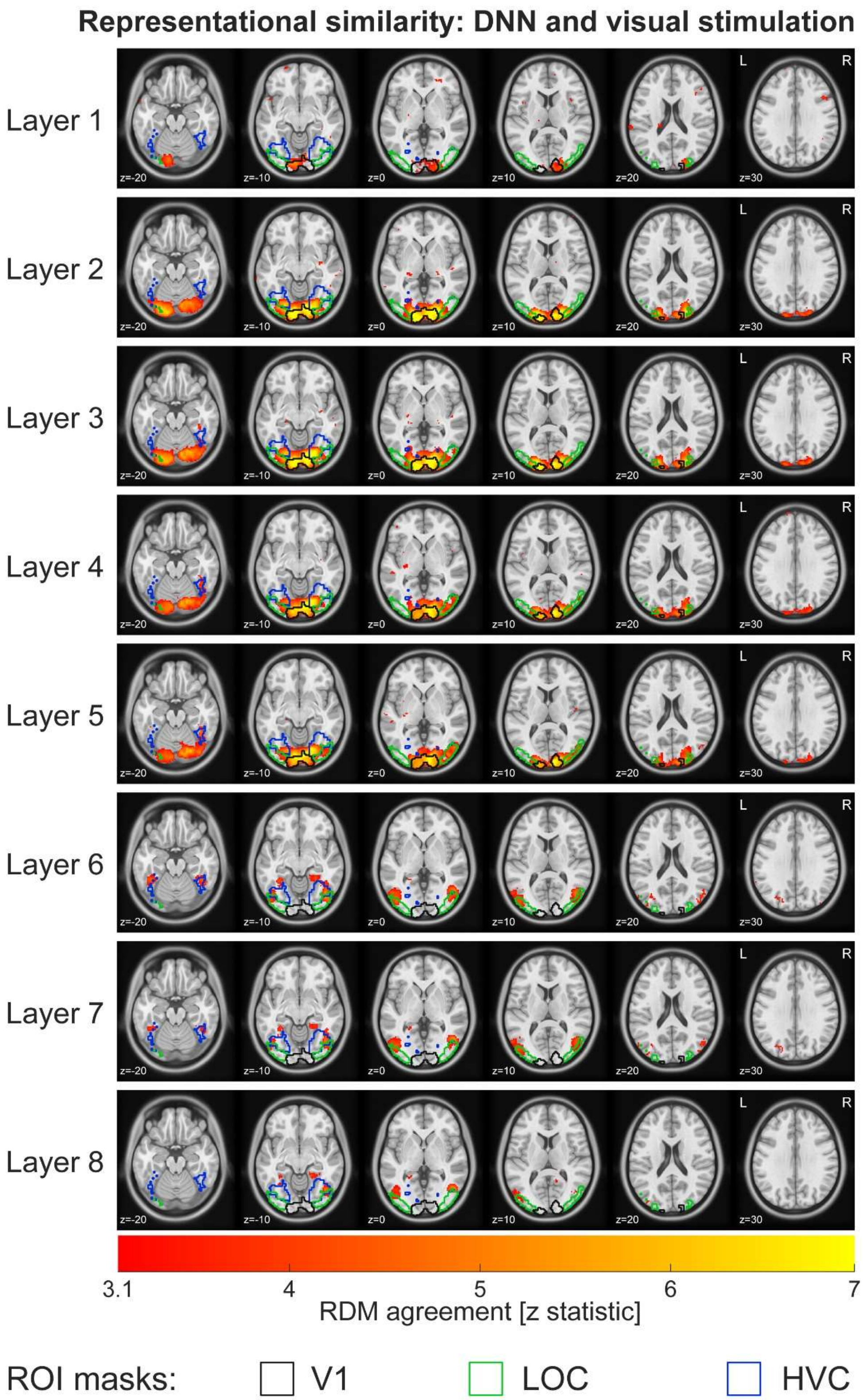

Figure depicting representational similarity analyses of visual responses showing that visual responses during the localizer were explained by a gradient of low-level to high-level visual features going up the ventral visual hierarchy. Early visual cortex (EVC) responses aligned more closely with early and intermediate DNN layers, indicative of low-level visual feature processing. Higher visual cortex (HVC) areas, like the fusiform gyrus, show a greater correlation with late DNN layers,

representing high-level visual feature processing. Red to yellow cluster indicate statistically significant correlations between the layer RDMs (layer 1-8 separately for each panel), thresholded at $z > 3.1$ (i.e., $p < 0.001$, uncorrected) of the RSA. ROI masks are illustrated as colored outlines with black = V1, green = LOC, and blue = HVC. A strong contrast between earlier convolutional layers (1-5) and later dense layers (6-8) is evident. Particularly early layers strongly map onto EVC, while later layers map onto late ventral visual areas. We also note that while our ROI definition does separate the ventral visual stream roughly into three separate stages early, intermediate and high-level visual cortex, our HVC definition does not include major anterior HVC clusters particularly correlating with late DNN layer representations. Critically, our results do not depend on the specific ROI mask definition, as larger ROI masks as those depicted here (Supplementary Figure 5) and an alternative ROI mask definition using all stimulus-driven voxels (Supplementary Figure 4) result in highly similar results as those obtained using the above depicted masks.

7) RE: Fig. 7A. To further substantiate the importance of high-level surprise, it would be useful to show reaction time modulation based on each of the DNN layers (not just layers 2 and 8)?

As discussed in more detail in reply to R2 major issue 1, neighboring DNN layers tend to correlate, hence resulting in issues of variance portioning. For this reason and explained in more detail in our reply to R2 (comment 1), we choose to focus on one low- and one high-level layer. However, in addition, R2 suggested including animacy category and word2vec dissimilarity in the RT analysis. Following the reviewer's suggestion, we added these regressors to the multiple regression RT analysis. Results, depicted below, now show that only animacy category surprise reliably explains RT increases, while layer 8 surprise no longer explains significant unique RT variance. Thus, in contrast to the fMRI BOLD results animacy category is the sole contributor to RT increases. Therefore, we removed the previous report of a significant scaling of RT with layer 8 surprise from the manuscript. For a summary of the associated test statistics see the table below. Thus, we believe that these updated results supersede the need for the inclusion of additional DNN layers in the RT analysis.

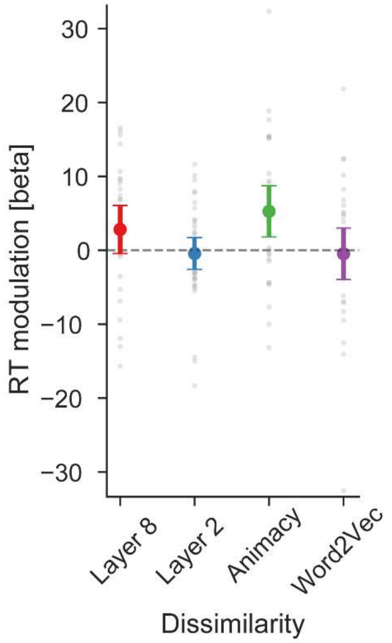

Figure. Behavioural response slowing correlates with animacy category surprise. Behavioral responses were slower when stimuli were of the unexpected animacy category. Neither high-level, low-level or word2vec surprise explained additional decrements in RT. Depicted are the slope coefficients (betas) regressing RT onto layer 8 (red), layer 2 (blue), animacy (green), and word2vec (purple) dissimilarity.

Contrast	Test statistic	P value	Effect size
Layer 8	$t_{(32)} = 1.96$	$p = 0.12$	$d_z = 0.34$
Layer 2	$t_{(32)} = -0.36$	$p = 0.97$	$d_z = -0.06$
Animacy category	$t_{(32)} = 3.24$	$p = 0.01$	$d_z = 0.56$
Word2Vec	$t_{(32)} = -0.28$	$p = 0.78$	$d_z = -0.05$

Table. Results of one sample t-tests contrasting RT modulations against zero. Only animacy category had a significant effect on RT scaling.

8) RE: (From the abstract) "…prediction errors may be computed at higher levels and propagate down the cortical hierarchy". It seems elsewhere in the paper, the idea that comes across (at least to me) is that high-level predictions are transmitted down to early visual cortex and the high-level errors are generated at this early stage. Perhaps this could be clarified in the text.

We agree with the reviewer that our original draft of the manuscript did not adequately clarify the interpretation of the results. Accordingly, we have updated our manuscript to elaborate more clearly on potential interpretations of our results in the context of predictive processing, as well as providing an alternative account emphasizing generic modulatory feedback signals (akin to the attention account previously discussed and refined in discussion with R2, major issue 4). As these adjustments involved changes in multiple parts of the Discussion section, we recommend reviewing the changes in the context of the manuscript. However, for completeness we also provide a list of changes here:

> "[...] one possible explanation for the observed results is that predictions allow the visual system to settle on a valid perceptual interpretation more quickly, as predictions match the bottom-up inputs. In contrast, unexpected input may require stronger (15) and more prolonged processing within the visual system to arrive at the best interpretation of the current inputs, potentially leading to the larger BOLD signal and superior decoding of stimulus representations observed here. Put differently, our results may suggest that the larger the difference between what is expected and what is observed, at a high-level, the more cycles of recurrent processing along the ventral visual stream may be necessary to solve object recognition." (line 421)
>
> "Moreover, merging prediction and attention accounts, we can derive another interpretation. Following prediction error computation in higher (visual) areas, a surprise signal could be relayed down the visual system. This surprise signal, instead of constituting a prediction, may be a scalar value reflecting the magnitude of the high-level surprise (or on a predictive coding account, the precision of the high-level prediction error) and result in a gain modulation of sensory neurons in earlier areas. This surprise signal could serve to allocate attention, facilitate learning, as well as update synaptic weights and thus promote encoding of statistical contingencies following highly surprising events. Sources of this modulatory feedback could be higher visual areas that compute high-level prediction errors or the perirhinal cortex, thereby closely linking high-level surprise with novelty detection and facilitating learning (54)." (line 556)
>
> "These results show a striking dissociation between the bottom-up tuning profile in early visual cortex and its modulation by high-level surprise. They suggest that visual surprise is computed at higher levels of the visual ventral stream and surprise signals are subsequently broadcast to earlier areas in the cortical hierarchy." (line 569)

We also adjusted the abstract, ensuring that we no longer imply that prediction errors are relayed down the visual hierarchy. The abstract now reads:

> "[...] or prediction errors may be computed at higher levels and the resulting surprise signal is broadcast to earlier areas in the cortical hierarchy." (line 26)

9) RE: scatter plots in Fig. 4B and 5D. (I may have missed it in the text but) Perhaps add an R value to help the reader assess the effect.

We chose to not include an r value for Fig. 4B, because as noted above, Fig. 4B merely reflects example data of one participant, which we now more clearly state in the figure text:

> "For illustration purposes, the graph at the bottom uses data from one example participant to depict how BOLD responses (example data from V1; ordinate) are modulated as a function of low-level (blue) and high-level (red) visual dissimilarity (abscissa)." (line 188)

We believe that reporting statistics for this illustration would lead to readers to over-interpret the example data of that one participant.

Concerning the results depicted in Fig. 5D, which are group level results, we believe that the inclusion of additional information, such as effect size metrics, is a valuable addition. However, it is important to consider which summary statistic is most informative. We would like to clarify that the regressions (Fig. 5D) are fit for each participant separately and the summary statistics reported in the text reflect second-level statistics performed on the obtained regression coefficients. Thus, here our analysis approach matches the common two-level analysis approach of standard fMRI analyses. While a common approach it makes the correct choice of effect size metric more difficult. Specifically, the critical group-level test is a one-sample t-test (or non-parametric equivalent) contrasting the obtained correlation coefficients against zero (i.e., no modulation). Therefore, we report in the associated Results text effect sizes as Cohen's d_z (i.e. within-subject effect size). However, we do feel that reporting Cohen's *d* in the scatter plot might confuse readers. In contrast the single-subject r values are not directly informative for the critical group-level test, thereby also not constituting the best indication of effect size. Finally, computing a group-level regression r value, with data averaged across subjects (as depicted in Fig5D) may also lead to incorrect conclusion, as the regressions were fit per subject. Thus, we now report both the average r value of the individual subject regressions, as well as Cohen'd in the Results text.

> "Results, shown in Figure 5D, show that the increased BOLD response to surprising stimuli follows a positive monotonic association across the dissimilarity spectrum in all three ROIs (V1: $t_{(32)} = 9.35$, $p < 0.001$, $d_z = 1.63$, mean $r = 0.09$; LOC: $t_{(32)} = 3.27$, $p = 0.004$, $d_z = 0.57$, mean $r = 0.03$; HVC: $t_{(32)} = 2.05$, $p = 0.049$, $d_z = 0.36$, mean $r = 0.02$)." (line 291)

References

Alink A, Blank H. Can expectation suppression be explained by reduced attention to predictable stimuli? NeuroImage. 2021; 231:117824.

Alink A, Abdulrahman H, Henson RN. Forward models demonstrate that repetition suppression is best modelled by local neural scaling. Nat Comm. 2018; 9:3854.

Guclu U, van Gerven MAJ. Deep Neural Networks Reveal a Gradient in the Complexity of Neural Representations across the Ventral Stream. J Neurosci. 2015 Jul 8;35(27):10005–14.

Kok P, Jehee JFM, de Lange FP. Less Is More: Expectation Sharpens Representations in the Primary Visual Cortex. Neuron. 2012 Jul;75(2):265–70.

Kumar S, Kaposvari P, Vogels R. Encoding of predictable and unpredictable stimuli by inferior temporal cortical neurons. J Cogn Neurosci. 2017; 29:1445–54.

Mehrer J, Spoerer CJ, Jones EC, Kriegeskorte N, Kietzmann TC. An ecologically motivated image dataset for deep learning yields better models of human vision. Proc Natl Acad Sci. 2021 Feb 23;118(8):e2011417118.

Meyer T, Olson CR. Statistical learning of visual transitions in monkey inferotemporal cortex. Proc Natl Acad Sci. 2011 Nov 29;108(48):19401–6.

Richter D, de Lange FP. Statistical learning attenuates visual activity only for attended stimuli. eLife. 2019 Aug 23;8:e47869.

Richter D, Ekman M, de Lange FP. Suppressed Sensory Response to Predictable Object Stimuli throughout the Ventral Visual Stream. J Neurosci. 2018 Aug 22;38(34):7452–61.

Richter D, Heilbron M, de Lange FP. Dampened sensory representations for expected input across the ventral visual stream. Oxf Open Neurosci. 2022 May 1;1:kvac013.

Yamins DLK, Hong H, Cadieu CF, Solomon EA, Seibert D, DiCarlo JJ. Performance-optimized hierarchical models predict neural responses in higher visual cortex. Proc Natl Acad Sci. 2014 Jun 10;111(23):8619–24.

Yan C, De Lange FP, Richter D. Conceptual associations generate sensory predictions. J Neurosci. 2023 Apr 14;JN-RM-1874-22.
