# Supplementary material for: High-level visual prediction errors in early visual cortex
Source: PLoS Biol. 2024 Nov 11;22(11):e3002829. doi: 10.1371/journal.pbio.3002829 (PMC11554119; doi:10.1371/journal.pbio.3002829)
Supplement: Attachment — Submitted filename: plos_response_to_reviewers_rev2.pdf [file pbio.3002829.s015.pdf]

Response to Reviewers

We thank the reviewers for the positive evaluation of our revised manuscript. Below we provide a reply to the remaining comments of R3. In brief, we agree with all suggestions made by the reviewer and have added the figures and text to the supplemental information and manuscript as suggested by the reviewer. Our replies are highlighted in blue.

Reviewer remarks:

Reviewer #1: The authors have addressed all points I had regarding their original submission.

Reviewer #2 (Arjen Alink): I would like to thank the authors for addressing all the issues I raised in such a thorough manner. With these changes made to the manuscript, I fully support its publication in the journal of PLOS Biology

Reviewer #3: I appreciate the effort of the authors in running the additional analyses as requested by the reviewers. Some of these additional analyses provided important information to interpret the results, but were not included in the manuscript. I recommend that they be included in the manuscript (not just the response to reviewers), to increase transparency; and it would be a quick revision as the analyses have already been done and could simply be cut and pasted into the manuscript. That is, I suggest the following to be added to the manuscript.

1) Please include the figure: "Representational similarity: DNN and visual stimulation" as a supplementary figure in the manuscript, which seems to only be contained in the Response to Reviewers. I think this figure is very useful to allow the reader to interpret the main results of the paper, e.g., it shows an overlap between early layers and the HVC.

Following the reviewer's suggestion, we now include the figure as S8 Fig.

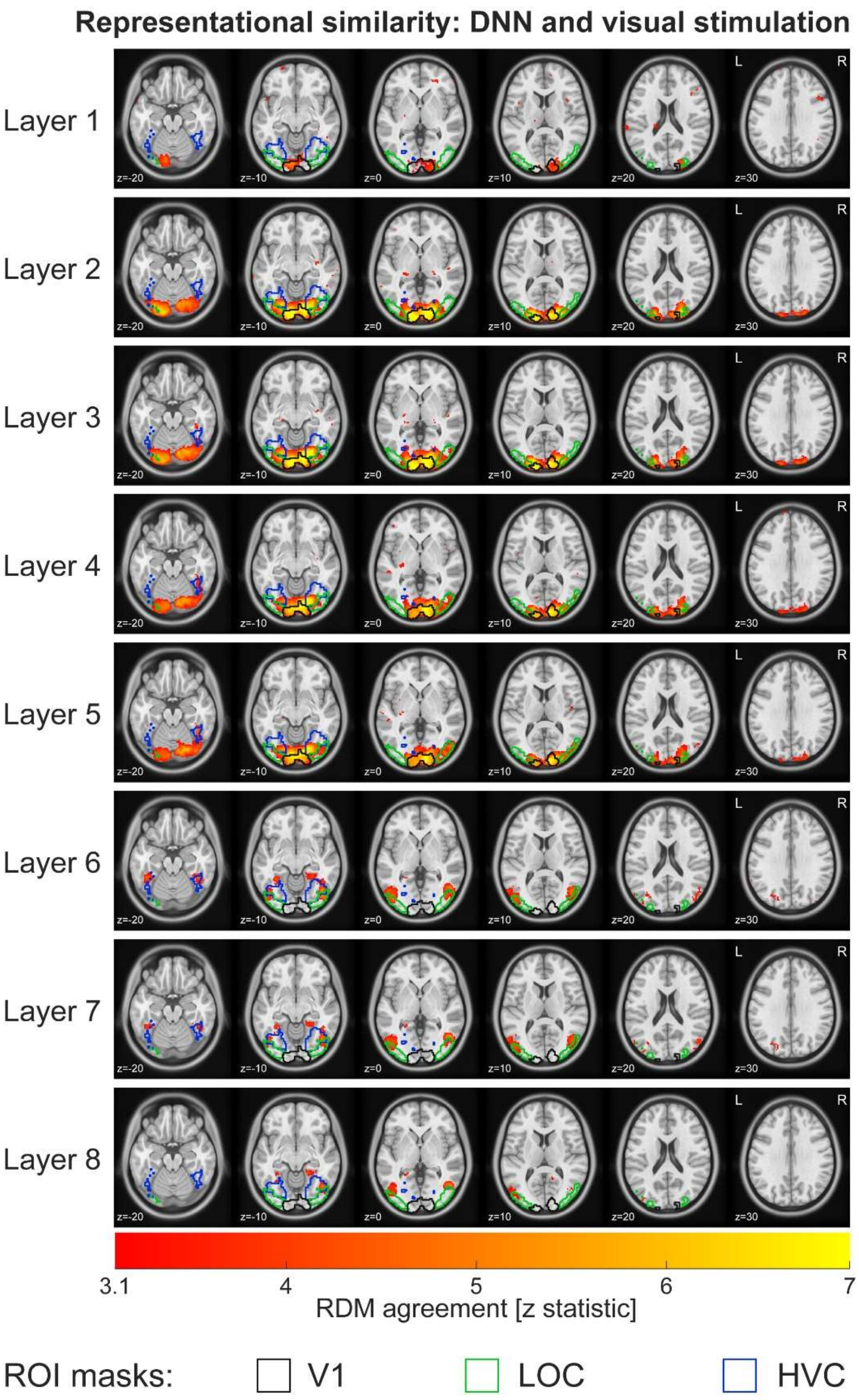

S8 Fig. Representational similarity analyses show that visual responses during the prediction-free localizer were explained by a gradient of low-level to high-level visual features going up the ventral visual hierarchy. Early visual cortex (EVC) responses align more closely with early and intermediate DNN layers, indicative of low-level visual feature processing. Higher visual cortex (HVC) areas, like the fusiform gyrus, show a greater correlation with late DNN layers, representing high-level visual feature processing. Red to yellow cluster indicate statistically significant correlations between the layer RDMs (layer 1-8 separately for each panel), thresholded at $z > 3.1$ (i.e., $p < 0.001$, uncorrected). ROI masks are illustrated as coloured outlines with black = V1, green = LOC, and blue = HVC. A strong contrast

between earlier convolutional layers (1-5) and later dense layers (6-8) is evident. Particularly early layers strongly map onto EVC, while later layers map onto late ventral visual areas. We also note that while our ROI definition does separate the ventral visual stream roughly into three separate stages corresponding to early, intermediate and high-level visual cortex, our HVC definition does not include major anterior HVC clusters particularly correlating with late DNN layer representations. Critically, our results do not depend on the specific ROI mask definition depicted, because larger ROI masks than those depicted here (see S5 Fig) and an alternative ROI mask definition using all stimulus-driven voxels (see S4 Fig) resulted in highly similar results as those obtained using the above depicted masks.

2) I suggest including a statement in the manuscript, clarifying that layers of visual DNNs tend to highly correlate so "ROI analysis results assessing the modulation of visual responses by surprise as indexed by feature dissimilarity across all 8 layers of the DNN" do not show reliable results. (RE: "Figure depicting the ROI analysis results assessing the modulation of visual responses by surprise as indexed by feature dissimilarity across all 8 layers of the DNN. ROIs in early visual (V1), intermediate (LOC) and higher visual cortex (HVC), show no reliable results. Error bars indicate the 95% within-subject confidence intervals. Gray dots denote individual subjects.") This is important to allow the reader to assess the limits of the approach.

We agree that mentioning this limitation of the approach is relevant. Indeed, we think that explaining that this is the reason why we focussed our key analyses on 2 layers (one for low-level and one for high-level visual features) is helpful for the readers to evaluate our approach. Therefore, we now include in the following statement in the manuscript's Discussion section when addressing potential limitations:

"A limitation of the present approach is that neighboring layers of visual DNNs tend to correlate and hence explain significant shared variance of visual representations (S8 Fig), thus contraindicating the inclusion of all layers into the same GLM. For this reason, our key analyses focused on two layers, layer 2 as low-level and layer 8 as high-level visual feature model."

3) Please include the following results in the manuscript which show that expected vs unexpected stimuli could not be decoded from the ROIs (which is surprising considering the main claims in the paper), i.e., (line 605 in response to reviewers) "…to obtain a mean decoding accuracy for expected and unexpected trials per participant. On average decoding of the object stimuli was well above chance (12.5%), but did not reliably differ between expected and unexpected stimuli in any of the three ROIs: HVC (Expected: 47.5%, ±3.0%; Unexpected: 46.9%, ±3.0% [mean, ±95%CI]), LOC (Expected: 63.1%, ±2.2%; Unexpected: 64.5%, ±2.6%), V1 (Expected: 73.1%, ±3.3%; Unexpected: 73.8%, ±3.3%).
ROI Test statistic P value
V1 $t(32) = -0.51$ $p = 0.613$
LOC $t(32) = -1.02$ $p = 0.315$
HVC $t(32) = 0.55$ $p = 0.588$

We agree with the reviewer that providing this information to the reader is a valuable addition. We now report the results of the decoding analysis as S5 Table.

**ROI**	**Expectation status**	**Decoding accuracy [mean ± 95% CI]**
V1	Expected	73.1% ± 3.3%

	Unexpected	73.8% ± 3.3%
LOC	Expected	63.1% ± 2.2%
	Unexpected	64.5% ± 2.6%
HVC	Expected	47.5% ± 3.0%
	Unexpected	46.9% ± 3.0%

S5 Table. Decoding of stimulus identity across expectation conditions. For each participant and within each ROI we extracted the single-trial parameter estimates (BOLD) from the localizer runs. On this data we trained an 8-class classifier, with the 8 classes corresponding to the 8 objects seen by the participant. Next, we tested the classifier on the single-trial parameter estimates from the main task runs. To improve SNR, we averaged the single-trial estimates for each object and expectation condition (expected or unexpected) within runs. Finally, we averaged the obtained decoding accuracies across runs to obtain a mean decoding accuracy for expected and unexpected trials per participant. On average decoding of the object stimuli was well above chance (12.5%), but did not reliably differ between expected and unexpected stimuli in any of the three ROIs (V1: $t_{(32)} = -0.51$, $p = 0.613$; LOC: $t_{(32)} = -1.02$, $p = 0.315$; HVC: $t_{(32)} = 0.55$, $p = 0.588$).
